# Molecular Networking-Guided Isolation of New Etzionin-Type Diketopiperazine Hydroxamates from the Persian Gulf Sponge *Cliona celata*

**DOI:** 10.3390/md19080439

**Published:** 2021-07-31

**Authors:** Reza Mohsenian Kouchaksaraee, Fengjie Li, Melika Nazemi, Mahdi Moridi Farimani, Deniz Tasdemir

**Affiliations:** 1 GEOMAR Centre for Marine Biotechnology (GEOMAR-Biotech), Research Unit Marine Natural Products Chemistry, GEOMAR Helmholtz Centre for Ocean Research Kiel, Am Kiel-Kanal 44, 24106 Kiel, Germany; rmohsenian@geomar.de (R.M.K.); fli@geomar.de (F.L.); 2 Persian Gulf and Oman Sea Ecological Center, Iranian Fisheries Science Research Institute, Agricultural Research, Education and Extension Organization (AREEO), 7916793165 Bandar Abbas, Iran; melikanazemi@pgoseri.ac.ir; 3 Department of Phytochemistry, Medicinal Plants and Drugs Research Institute, Shahid Beheshti University, Evin, 1983969411 Tehran, Iran; m_moridi@sbu.ac.ir; 4 Faculty of Mathematics and Natural Sciences, Kiel University, Christian-Albrechts-Platz 4, 24118 Kiel, Germany

**Keywords:** Persian Gulf, marine sponge, *Cliona celata*, molecular networking, diketopiperazine, etzionin, clioetzionin A, clioetzionin B

## Abstract

The Persian Gulf is a unique and biologically diverse marine environment dominated by invertebrates. In continuation of our research interest in the chemistry and biological activity of marine sponges from the Persian Gulf, we selected the excavating sponge *Cliona celata* for detailed metabolome analyses, in vitro bioactivity screening, and chemical isolation studies. A UPLC-MS/MS (MS^2^) molecular-networking-based dereplication strategy allowed annotation and structural prediction of various diketopiperazines (DKPs) and etzionin-type diketopiperazine hydroxamates (DKPHs) in the crude sponge extract. The molecular-networking-guided isolation approach applied to the crude extract afforded the DKPH etzionin (**1**) and its two new derivatives, clioetzionin A (**2**) and clioetzionin B (**3**). Another new modified DKP (**4**) was identified by MS/MS analyses but could not be isolated in sufficient quantities to confirm its structure. The chemical characterization of the purified DKPHs **1**–**3** was performed by a combination of 1D and 2D NMR spectroscopy, HRMS, HRMS/MS, and [α]_D_ analyses. Compounds **1** and **2** exhibited broad antibacterial, antifungal, and anticancer activities, with IC_50_ values ranging from 19.6 to 159.1 µM. This is the first study investigating the chemical constituents of a *C. celata* specimen from the Persian Gulf. It is also the first report of full spectroscopic data of etzionin based on extensive spectroscopic analyses.

## 1. Introduction

Iran covers the entire northern coastline of the Persian Gulf, a marginal sea of the Indian Ocean. The hot and dry nature of the geographical area, where evaporation of water exceeds the freshwater input tenfold, as well as the separation from open waters by the Strait of Hormuz, makes the Persian Gulf a hypersaline environment. The Persian Gulf is a shallow sea with high temperature fluctuations, ranging from 12 to 35 °C, representing a significant stress factor for marine flora and fauna [1,2]. Despite all these harsh environmental conditions, plus additional anthropogenic pressures, such as busy shipping lines, the Persian Gulf has a highly diverse fauna dominated by coral and sponge communities [3]. However, only a few chemical studies have investigated the chemical composition or the biological activity of these organisms.

The sponge genus *Cliona* (family Clionidae) comprises about 80 species that are common in the Mediterranean Sea and the Atlantic, Indian, and Pacific Oceans [4,5]. *Cliona* species are burrowing sponges that live on a variety of calcareous substrates, such as rocks, shells, coral, and coralline algae, where they play an important role in the erosion of the calcium carbonate layer [6]. Chemically, the genus has been little studied, but the few studies performed on this genus so far have revealed the presence of diverse natural product classes, such as steroids, alkaloids, and peptides [7,8,9,10]. Peptidic constituents of the genus *Cliona* include clionamide, the major metabolite of *C. celata*; tetraacetyl clionamide; celenamides A, B, and E; and cyclo-(l-Pro-l-Tyr) diketopiperazine (DKP) [10,11,12,13,14], reported from *Cliona* sponges collected from various geographical regions, such as Argentina, Canada, Spain, Morocco, and Thailand [7,8,9,10,11,12,13,14]. Only two *Cliona* species, namely *C. celata* and *C. vastifica*, have been reported from the fauna of the Persian Gulf [15], but they have not been subjected to any chemical or bioactivity study so far.

As part of our continuing project on the chemistry of the sponges of the Persian Gulf [16], we selected *C. celata* for in-depth investigations. Using an HRMS/MS-based molecular networking strategy and interpretation of the MS/MS spectra, we annotated diketopiperazines (DKPs) and etzionin-type diketopiperazine hydroxamate derivatives (DKPHs) in the crude extract. The presence of DKPHs in the molecular networking was further verified by targeted isolation and structural elucidation of the major compounds based on extensive HRMS/MS, NMR spectroscopy, and specific rotation ([α]_D_) values. Here, we report the bioactivity and chemical profiling of the *C. celata* crude extract and detailed analyses of the MS/MS fragmentation pattern of DKPs and DKPHs, followed by purification, characterization, and bioactivity assessment of three DKPHs (**1**–**3**) isolated from this sponge.

## 2. Results

### 2.1. Bioactivity Profiling of the Crude Sponge Extract

The sponge material was extracted with water, MeOH, and DCM. The organic extracts were combined and screened for in vitro biological activities, including antimicrobial activity against the bacterial *Enterococcus faecium*, methicillin-resistant *Staphylococcus aureus* (MRSA), *Klebsiella pneumoniae*, *Acinetobacter baumannii*, *Pseudomonas aeruginosa*, and *Escherichia coli* (ESKAPE) panel, antifungal activity (against *Candida albicans* and *Cryptococcus neoformans*), and cytotoxicity against six cancer cell lines (human breast MDA-MB231, malignant melanoma A375, colorectal adenocarcinoma HT29, colon cancer HCT116, liver cancer Hep G2, and lung carcinoma A549) and the non-cancerous human keratinocyte cell line HaCaT. The crude extract was inactive at the test concentration of 100 µg/mL in all assays.

### 2.2. MS/MS Molecular-Networking-Based Dereplication of the Crude Extract

To unlock the chemical diversity of the crude extract, the MS/MS data generated by a tandem UPLC-QToF-MS spectrometer in positive-ion mode were analyzed by MZmine 2.53 to pre-process data [17] and then uploaded to the publicly available Global Natural Product Social Molecular Networking (GNPS; www.gnps.ucsd.edu) platform [18]. The resulting molecular network was visualized by the software Cytoscape 3.7.1, leading to the observation of 80 nodes in 11 clusters (at least 2 nodes per cluster) (Supplementary Figure S1). Since the automated comparison of experimental MS/MS spectra with spectral libraries on GNPS did not allow annotation of any known compounds, a manual dereplication was performed by searching for the predicted molecular formulae of detected ion features in several databases, such as Dictionary of Natural Products, MarinLit, Reaxys, and SciFinder, and performing a similarity-based comparison between experimental MS/MS spectra and those predicated by in silico fragmentation methods, e.g., CFM-ID.

**Figure 1:**
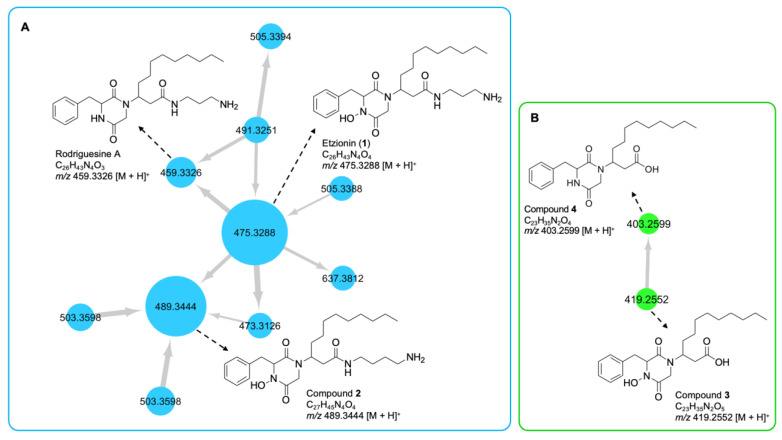
Molecular clusters (**A**,**B**) observed in the crude extract of *C. celata*. The node size represents the peak area of features. Numbers within the nodes indicate parent ions, and the edge thickness represents the cosine similarity of the nodes.

**Figure 2:**
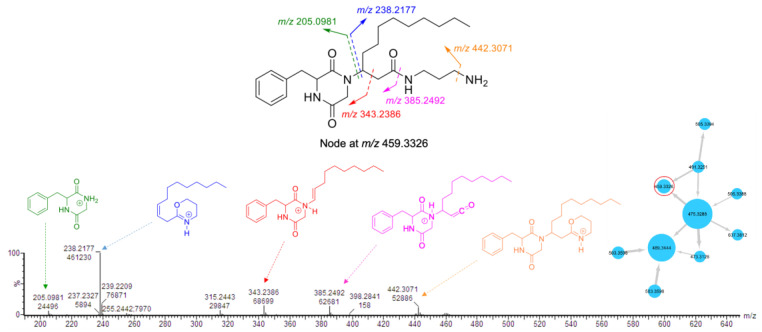
Fragmentation pattern of rodriguesine A (the node at *m*/*z* 459.3326 in cluster **A**).

The node with parent ion *m*/*z* 459.3326, belonging to the largest cluster (**A**) in the molecular network of the crude extract (Supplementary Figure S1, Figure 1), was annotated as rodriguesine A, an antibacterial modified DKP isolated from the ascidian *Didemnum* sp., by comparing its molecular formula (C_26_H_43_N_4_O_3_) and experimental MS/MS fragmentation patterns with those reported [19]. The experimental HR-MS/MS spectrum of this node displayed product ions at *m*/*z* 205.0981, 238.2177, and 343.2386, corresponding to a DKP moiety composed of a phenylalanine and a glycine residue, an oxazine ion, and a diketopiperazinium ion, respectively (Figure 2). Neutral losses of C_3_H_10_N_2_ (1,3-diaminopropane) and NH_3_ allowed the formation of additional product ions at *m*/*z* 385.2492 and 442.3071 (Figure 2), respectively. It should be noted that the node size corresponds to the molecule’s relative abundance, calculated from its LC-MS peak area.

**Figure 3:**
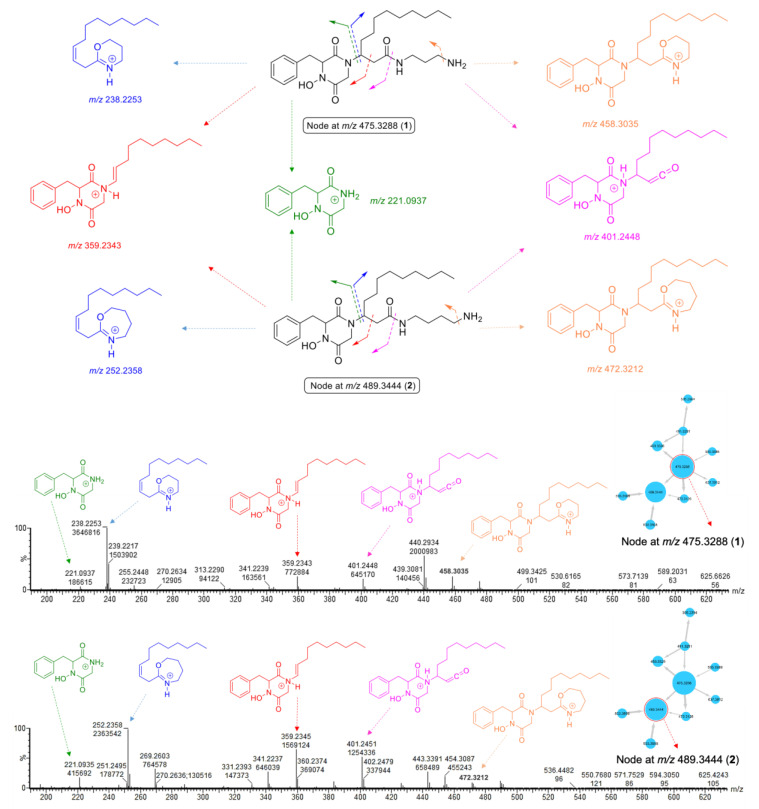
Fragmentation pattern of the nodes at *m*/*z* 475.3288 (**1**) and *m*/*z* 489.3444 (**2**) in cluster **A**.

The largest node in cluster **A** (*m*/*z* 475.3288) was closely related to rodriguesine A, indicated by the thick edge between the two nodes (Figure 1A), and based on its molecular formula (C_26_H_43_N_4_O_4_), it was predicted to be a hydroxylated derivative of rodriguesine A. It yielded the same oxazine fragment ion at *m*/*z* 238.2253 as rodriguesine A (Figure 2 and Figure 3) and the same characteristic product ions with 16 mass units higher than those of rodriguesine A, i.e., *m*/*z* 401.2448 [M-C_3_H_10_N_2_]^+^, 221.0937 (diketopiperazine hydroxamate moiety), and 359.2343 (diketopiperazinium hydroxamate moiety), further supporting a hydroxy substitution on the DKP moiety (Figure 3). Hence, the node *m*/*z* 475.3288 was annotated as etzionin (**1**), a DKP hydroxamate with antifungal activity obtained from an unidentified red tunicate collected in the Gulf of Eilat (the Red Sea) [20]. The complete chemical structure of etzionin (**1**) was further confirmed by purification and structure elucidation via spectroscopic methods (see Section 2.3).

The second-largest node at *m*/*z* 489.3444 was also linked to etzionin by a thick edge (Figure 1A) and showed a similar MS/MS fragmentation pattern (Figure 3). However, the oxazine ion was replaced by an oxazepane ion at *m*/*z* 252.2358 in its MS/MS spectrum. This suggested the attachment of a diaminobutane unit, instead of a diaminopropane unit, to the amide carbon (C-1′) on the side chain (Figure 3). Hence, the node at *m*/*z* 489.3444 was annotated as a new etzionin derivative (compound **2**) with an extra CH_2_ unit on the linear side chain. The structure of **2** was further ascertained by purification and structure elucidation (see Section 2.3).

The remaining seven nodes in cluster **A** were annotated as putatively new derivatives of rodriguesine A and etzionin (Figure 1A; Supplementary Table S1) by interpretation of their MS/MS fragmentation patterns and comparison with those of the parent compounds. However, except for compounds **1** and **2**, we could not purify them in sufficient amounts for structure elucidation.

**Figure 4:**
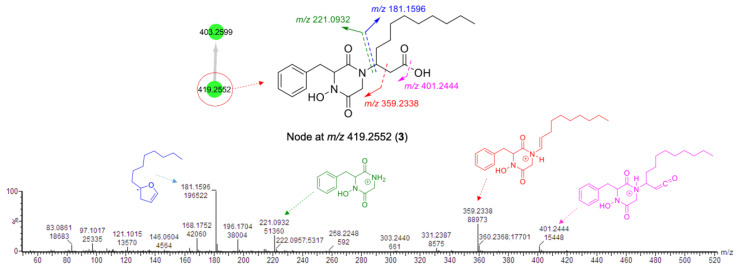
Fragmentation pattern of the node at *m*/*z* 419.2552 (**3**) from cluster **B**.

**Figure 5:**
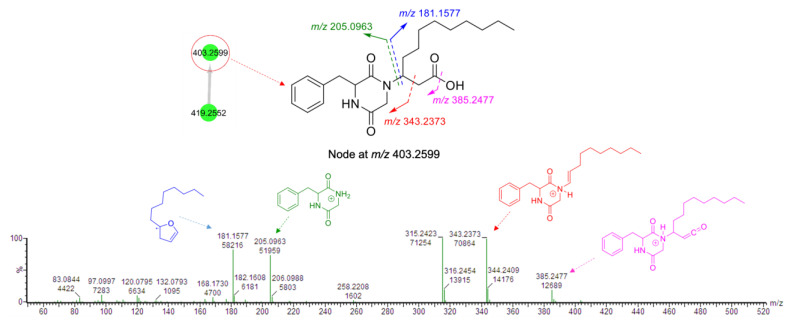
Fragmentation pattern of the node at *m*/*z* 403.2599 found in cluster **B**.

The small molecular family **B** (Figure 1B) that comprised two nodes was also annotated as DKPs based on the nodes’ characteristic fragmentation patterns (Figure 4 and Figure 5). As discussed below, the missing diamine moiety on the side chain led to the separation of cluster **B** from cluster **A**. The node at *m*/*z* 419.2552 showed a fragmentation pattern similar to that of etzionin (*m*/*z* 221.0932, 359.2338, and 401.2444). However, the MS/MS spectrum of this node contained an intense ion at *m*/*z* 181.1596, corresponding to the loss of the whole DKP moiety (Figure 4). Aided by the elemental composition analyses (C_23_H_35_N_2_O_5_), the node at *m*/*z* 419.2552 was putatively annotated to be a new derivative of etzionin, where the amide group was replaced by a terminal carboxylic acid. Its structure (**3**) was further verified after purification and spectroscopic analyses (see Section 2.3).

The second node in cluster **B** (*m*/*z* 403.2599, Figure 1B) had a fragmentation pattern comprising the product ion *m*/*z* 181.1577 similar to that of **3,** as well as three product ions (*m*/*z* 205.0963, 343.2373, and 385.2477) that were 16 mass units lower than that observed for **3** (Figure 5). Considering its molecular formula, C_23_H_35_N_2_O_4_, this compound (**4**) was annotated as a new derivative of **3** without the hydroxamate functional group. We failed to purify compound **4** in sufficient quantities for spectroscopic analyses. However, it is a new compound based on MS/MS fragmentation.

### 2.3. Purification and Structure Elucidation of DKPHs

Initial UPLC-HRMS/MS profiling of the crude organic extract from *C. celata* revealed the presence of diverse new DKPH analogues. The fractionation of the crude extract by semi-preparative RP-HPLC guided by molecular networking analyses yielded nine fractions (F1–F9), of which F3 and F4 were found to be the main reservoirs of DKPH analogues, which was also supported by ^1^H NMR analyses (Supplementary Figure S3). Further RP-HPLC purification of F3 and F4 afforded DKPHs **1**–**3** (Figure 6). Their enantiopurity was checked by RP-HPLC-DAD equipped with an analytical chiral column. DKPHs were characterized by interpretation of their NMR, HR-ESIMS, and MS/MS data and [α]_D_ values (Supplementary Figures S4–S26).

Compound **1** was obtained as an optically active yellow film ([α]^20^_D_ +30.3, *c* 0.1, CHCl_3_). The molecular formula C_26_H_43_N_4_O_4_ was deduced from its HR-ESIMS (*m*/*z* 475.3285, [M + H]^+^) spectrum (Supplementary Figure S12), requiring eight degrees of unsaturation (DoU). As mentioned above, molecular-networking-based dereplication annotated compound **1** as etzionin, a known DKPH that was initially reported in 1989 from a Red Sea tunicate without MS data [20]. Its ^1^H NMR spectrum [20] showed only broad, poorly defined resonances in a mixture of CDCl_3_ and CD_3_OD. Because of the lack of full NMR data in the literature, we decided to acquire the full set of 1D and 2D NMR spectra of **1** and assign its complete NMR resonances. The 1D NMR spectra (Table 1 and Table 2) readily indicated the presence of three amide carbonyl groups, *δ*_C_ 167.3 (C-2), 162.2 (C-5), and 173.2 (C-1′), and a monosubstituted benzene ring with resonances at *δ*_H_/*δ*_C_ 7.20/131.5 (H-9, H-13/C-9, C-13), 7.30/129.7 (H-10, H-12/C-10, C-12), 7.30/128.7 (H-11/C-11), and *δ*_C_ 136.1 (C-8). As seven of the eight DoU were accounted for by three carbonyls and one benzene ring, **1** had to be monocyclic. 

**Figure 7:**
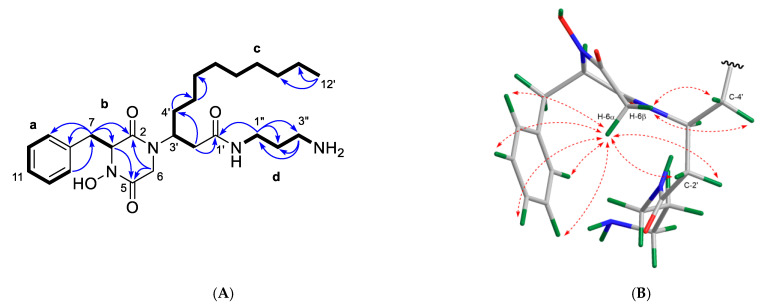
(**A**) Key COSY (bold) and HMBC (H→ C, arrows) correlations observed for compound **1**. (**B**) Key NOESY correlations (dashed line) shown on the Chem3D-optimized model of **1**.

As shown in Figure 7, the ^1^H-^1^H COSY spectrum of **1** indicated the presence of four spin systems (**a**–**d**). The ^1^H-^13^C HMBC correlations observed from H_2_-7 to C-2, C-3, C-8, and C-9 and between H-13/C-7 connected the spin systems **a** and **b**, forming the phenylalanine residue. Additional correlations observed in the HMBC spectrum between H_2_-6/C-2, H_2_-6/C-5, and H-3/C-5 identified a glycine residue and further constructed the diketopiperazine (DKP) moiety. Key HMBC correlations from H_2_-2′ to C-1′ and C-4′ plus additional correlations shown in Figure 7 suggested the presence of a linear alkyl β-amino acid residue, while a diagnostic NOESY correlation between H_2_-6 and H-3′ confirmed that it was connected to the DKP unit by a direct C–N bond, i.e., C-3–N-1 [20]. Similarly, ^1^H–^1^H COSY correlations between H_2_-1″, H_2_-2″, and H_2_-3″ (**d**) plus the HMBC correlations between H_2_-1″/C-1′, H_2_-1″/C-2″, and H_2_-1″/C-3″ allocated the mono-acylated diamine moiety, as shown in Figure 7. A detailed analysis of the MS/MS spectrum of **1** assigned the remaining hydroxy group to N-4, evident by the characteristic product ion at *m*/*z* 221.0937 (Figure 3), thereby leading to the planar structure of **1**.

Compound **1** contains two stereocenters (C-3 and C-3′). The NOESY correlations between relevant protons on the Chem3D-optimized model of **1** assisted the assignment of the relative stereochemistry of **1** (Figure 7B). The missing ^3^*J* ^1^H-^13^C HMBC correlation between one of the geminal protons of C-6 (*δ*_H_ 2.51) and *δ*_C_ 167.3 (C-2) suggested a dihedral angle close to 90° between them [21,22], hence assigning the ^1^H resonance at *δ*_H_ 2.51 to be axial (H-6α) and *δ*_H_ 3.47 to be equatorial (H-6β) (Figure 7B). The NOE correlation between H-6α and the benzene protons (*δ*_H_ 7.20–7.28) suggested H-3 to be β-oriented (Figure 7B) and established the relative configuration at C-3 as R*. This also explained the upfield shift of H-6α compared with H-6β due to the diamagnetic anisotropy effect of the benzene ring (Figure 7B; Table 1). The observed NOE correlations between H-6α/H-2′ (*δ*_H_ 2.16) and H-6β/H_2_-4′ (*δ*_H_ 1.51 and 1.57) suggested H-3′ to be α-oriented (Figure 7B), thereby establishing the relative configuration of C-3′ as S*.

Compound **1** exhibited a positive optical rotation value in both CHCl_3_ ([α]_D_ +30.3, *c* 0.1) and MeOH ([α]_D_ +23.0, *c* 0.1). The original publication that reported etzionin from an unidentified Red Sea tunicate [20] did not include any stereochemical assignment or an [α]_D_ value. However, its diacetyl derivative was described to display an [α]_D_ value of +14 (*c* 0.1, MeOH) [20]. A subsequent study that aimed to elucidate the absolute configuration of etzionin also omitted the [α]_D_ value of the natural product isolated from a New Caledonian ascidian *Didemnum rodriguesi* [23]. Notably, the semi-synthetic etzionin derivative 4-dehydroxy-3″-acetyletzionin displayed an [α]_D_ value of −51.9 (*c* 0.08, CHCl_3_) [23]. The specific rotations of (3*S*,3′*R*)-4-dehydroxy-3″-acetyletzionin and (3*R*,3′*S*)-4-dehydroxy-3″-acetyletzionin by total synthesis had opposite signs, with [α]_D_ values of −53.1 (*c* 0.06, CHCl_3_) and +53.5 (*c* 0.11, CHCl_3_), respectively [23]. Due to ambiguity of the previous reports, the novelty and absolute stereochemistry of **1** remain somehow uncertain. Our study represents the first complete NMR data assignment of etzionin based on extensive 2D NMR spectroscopy and MS/MS analysis.

**Figure 8:**
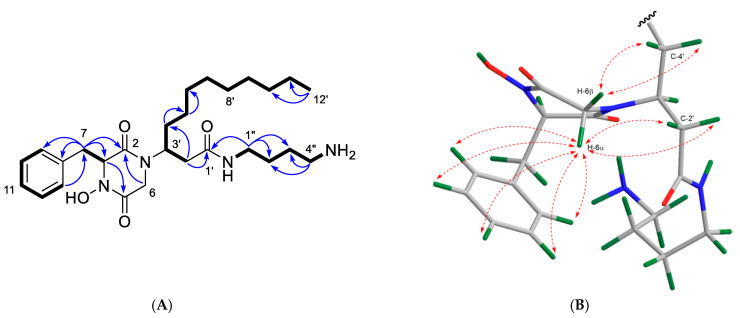
(**A**) Key COSY (bold) and HMBC (H→C, arrows) correlations observed for compound **2**. (**B**) Key NOESY correlations (dashed line) shown on the Chem3D-optimized model of **2**.

Compound **2** was obtained as a yellowish film. The molecular formula C_27_H_45_N_4_O_4_ (*m*/*z* [M + H]^+^ 489.3443) was deduced from its HR-ESIMS spectrum (Supplementary Figure S19). Analysis of the ^1^H and ^13^C NMR data revealed a high similarity between compounds **1** and **2**. Indeed, the only difference between the two compounds was the presence of an extra CH_2_ unit in the diamine moiety in **2**, which was supported by the HR-ESIMS data. The same DKPH core structure was readily established from the 1D and 2D NMR data of **2** (Table 1 and Table 2; Figure 8A). Key COSY cross-peaks between H_2_-1″/H_2_-2″, H_2_-2″/H_2_-3″, and H_2_-3″/H_2_-4″ and the HMBC correlations observed between H_2_-1″/C-1′, H_2_-1″/C-2″, and H_2_-1″/C-3″ and between H_2_-4″/C-2″ and H_2_-4″/C-3″ (Figure 8A) provided evidence that the butane-1,4-diamine group (at C-1′) replaced the propanediamine residue in **1**. The NOE cross-peaks observed between H-6α and benzene protons H_1_-9-H_1_-13, H-6α/H-2′, and H-6β/H_2_-4′ (Figure 8B) plus the same sign of the [α]_D_ value (+21.3 *c* 0.1, CHCl_3_) indicated that **2** has the same relative stereochemistry as **1**. We named the new compound **2** clioetzionin A.

**Figure 9:**
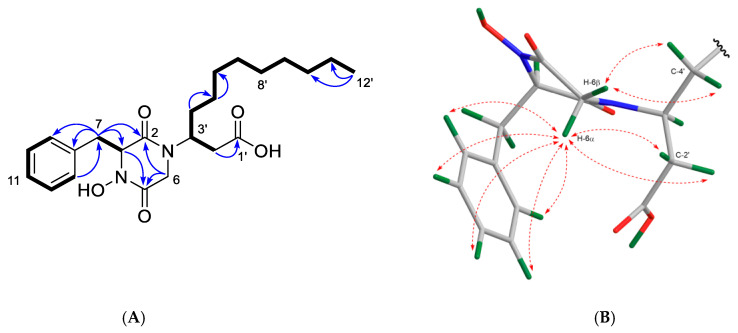
(**A**) Key COSY (bold) and HMBC (H→C, arrows) correlations observed for compound **3**. (**B**) Key NOESY correlations (dashed line) shown on the Chem3D-optimized model of **3**.

Compound **3** was purified as a yellowish film. Its molecular formula was readily assigned as C_23_H_35_N_2_O_5_ by its HR-ESIMS spectrum, which suggested the presence of eight DoU (Supplementary Figure S26). Its 1D and 2D NMR spectral data not only indicated its close resemblance with **1** and **2** (Table 1 and Table 2) but also confirmed the absence of the diamine residue at C-1′ in **3**. Key HMBC correlations between H_2_-2′ and C-1′ plus the downfield resonance of C-1′ (*δ*_C_ 175.8) confirmed the presence of a carboxylic acid function at C-1′ (Figure 9A) in **3**. The same NOE correlations (Figure 9B) and the same sign of the [α]_D_ value obtained for **3** (+15.3 *c* 0.1, CHCl_3_) assigned the same relative stereochemistry as found in **1** and **2**. Compound **3** was named clioetzionin B.

### 2.4. Bioactivity of Compounds **1** and **2**

Due to the low amount of compound **3** isolated, only **1** and **2** were tested in vitro against three bacterial strains, namely MRSA, *E*. *faecium*, and *E*. *coli*; two fungal strains, *C. albicans* and *C. neoformans*; two cancer cells lines, A375 and HCT-116; and the non-cancerous human keratinocyte cell line HaCaT. Compound **1** showed inhibitory activity against *E*. *faecium* and *C. neoformans*, with IC_50_ values of 19.6 and 22.6 µM, respectively (Table 3). Two- and fivefold lower activity was observed against MRSA and *C. albicans*, respectively. Compound **1** showed a moderate and equipotent activity against the cancer cell lines A375 and HCT116. However, the IC_50_ values were practically identical to those obtained against the non-cancerous HaCaT cell line, indicating a general toxicity for **1**. A similar activity profile was displayed by compound **2** against all test strains and cell lines (Table 3). The weakest activity was observed against the Gram-negative bacterium *E. coli* by compounds **1** and **2**, with IC_50_ values of 159.1 and 152.9 µM, respectively.

## 3. Discussion

One difficulty in classical natural product research is the re-isolation of already known compounds. Therefore, it is crucial to implement efficient dereplication strategies from the earliest (crude extract) stage to focus on new metabolites. In a previous study, we investigated the metabolome of the crude extract of the Persian Gulf sponge *Axinella sinoxea* by a combined MS/MS-based molecular networking and ^1^H NMR spectroscopy approach. This guided and facilitated the isolation and structure elucidation of eight metabolites, including a new DKP [16]. In the continuation of our project on the demosponges of the Persian Gulf, we now investigated the secondary metabolome of *C. celata*, a perforating sponge collected from the same site as *A. sinoxea.* Herein, we used, successfully, an MS/MS-based molecular networking dereplication strategy for (a) the identification of the chemical profile of the crude sponge extract, (b) the chemical structure prediction of many DKPs and DKPHs through their MS/MS fragmentation patterns, and (c) the targeted isolation of the DKPHs **1**–**3**. The molecular-networking-based untargeted metabolomics approach indicated the presence of other putatively new DKP derivatives in the crude extract. However, we were unable to purify them due to their minor quantity.

Excavating (perforating, burrowing, and boring) sponges live in calcium carbonate cavities, which they create through mechanical and chemical processes [24]. Various invertebrates, such as sponges, tunicates, cnidarians, crustaceans, and mollusks, use foreign materials as a cover to protect or mask their bodies [25]. Some sponges are able to incorporate these particles into their bodies to strengthen their skeletons [26]. *Cliona* sp. develop excavating or boring forms living on calcareous substrates, such as rocks, shells, coral, and coralline algae [6]. *Cliona celata* is an excavating sponge that develops a large massive form, i.e., the wall-shaped sponge covered with characteristic flattened papillae [27]. Chemical constituents of *Cliona* sp. are believed to contribute to its chemical defense, playing a role in deterring generalist fish (antipredatory), inhibiting larval settlement (antifouling), and competing for space with corals (allelopathy) [28]. Clionapyrrolidine A isolated from *C. tenuis* is able to kill the tissues of the stony coral *Acropora palmata* [29].

*Cliona* species have received limited interest for their chemical constituents. So far, steroids, including unusual amino- and halogen-bearing steroids, fatty acids, alkaloids, and peptidic compounds, have been reported from this genus [7,8,9,10]. Barry et al. reported the GC-MS-based identification of fatty acids (palmitic, palmitoleic, stearic, oleic, and linoleic acid) and brassicasterol, campesterol, cholesterol, β-sitosterol, Δ^7^-avenasterol, stigmasterol, Δ^5^-stigmasterol, and Δ^7^-stigmasterol type of steroids from *C. viridis* collected from the Atlantic coast of Morocco [7]. Polyhalogenated steroids clionastatins A and B with potent antitumor activity were reported from *C. nigricans* by Fattorusso et al. [30]. Autophagy-modulating aminosteroids clionamines A–D were isolated from *C. celata* sourced from South Africa [31]. There have been several reports on peptide-type compounds of the genus *Cliona* [10,11,12,13,14]. The first peptidic compounds tetraacetyl clionamide and clionamide with antibacterial activity were reported in the late 1970s from Canadian *C. celata* specimens [32]. Further linear peptide alkaloids celenamides A–D were subsequently obtained from the same sponge [12,14]. The tripeptide alkaloid celenamide E was obtained from *C. chilensis* and showed moderate antibiotic activity against Gram-positive bacteria (*S. aureus*, *Micrococcus luteus*, *Bacillus subtilis*, and *Enterococcus faecalis*) [13]. Storniamides A–D sourced from a Patagonian *Cliona* sp. displayed moderate activity against the same panel of Gram-positive bacteria [33]. A comprehensive study performed on *C. patera* by Sawangwong et al. led to the purification of two macrolides, namely tetillapyrone and nortetillapyrone; two alkaloids, namely maleimide 5-oxime and 3-methylmaleimide-5-oxime; and the DKP cyclo-(l-Pro-l-Tyr) from *C. patera* [10].

DKPs, the smallest cyclic peptides, are an abundant class of biologically active natural products [34]. They have been obtained from various marine macroorganisms, e.g., tunicates, sponges, gorgonians, mussels, sea urchins, red algae, mangroves, and sediment or mud. Such a wide occurrence of natural products in taxonomically unrelated phyla is often indicative of a microbial origin. Indeed, DKPs have been reported from marine fungi, yeast, and bacteria, including actinobacteria [34,35,36,37]. There have been many reports on the isolation of DKPs from sponges [16,37]. DKP hydroxamates (DKPHs) are relatively uncommon in nature and have been isolated from tunicates and mollusks with promising antifungal activity [20,36]. Etzionin belongs to the DKPH family and was originally reported from an unidentified tunicate from the Gulf of Eilat (the Red Sea) [20]. Etzionin is structurally closer to rodriguesic acids and rodriguesins A and B that were isolated from the gastropod mollusk *Pleurobranchus areolatus* and the ascidian *Didemnum* sp., respectively [19,36]. In this study, for the first time, we isolated and characterized etzionin, as well as its two new derivatives, from a marine sponge. It would be intriguing to adopt a microbial approach to investigate the true natural origin of compounds **1**–**3** in the future.

DKPs have been shown to exert diverse biological activities, such as antimicrobial, anti-inflammatory, anticancer, antiviral, antitumor, antiproteolytic, cytotoxic, phytotoxic, and insecticidal [34], while DKPHs are mainly known for their antimicrobial activities [19,20,21]. The initial report on etzionin [20] stated its promising antifungal activity against *C. albicans*, with an MIC value of 6.3 µM. Rodriguesins A and B that were isolated as a mixture exhibited moderate antimicrobial activity against *S. aureus* (ATCC6538 and ATCC259223) and *E. coli* (ATCCNTCC861 and ATCC259222), with MIC values ranging from 22.6 µg/mL to 125.0 µg/mL [19]. DKPHs **1** and **2** isolated in the current study showed broad-spectrum antimicrobial activity against human pathogenic bacteria and fungi, which is in agreement with the literature. Plinabulin, the synthetic *tert*-butyl analog of the marine fungal DKP halimide, is currently undergoing phase III clinical trials against cancer [38,39]**.** Inspired by this fact, we tested compounds **1** and **2** against the malignant melanoma cell line A375 and the colon cancer cell line HCT116. We observed moderate anticancer activity for both DKPHs, but they also inhibited the growth of non-cancerous HaCaT cells, indicating that they have no selective toxicity toward cancer cell lines. Etzionin has been shown to exert moderate anticancer activity against the P388 murine leukemia cell line, with an IC_50_ value of 21 µM [20], but its general toxicity has not been assessed. Interestingly, we did not observe any bioactivity with the crude organic extract of the sponge, possibly due to the presence of the DKPHs in low amounts.

## 4. Materials and Methods

### 4.1. General Experimental Procedures

HR-MS/MS data of the crude extract were obtained on a Waters Xevo G2-XS QTof Mass Spectrometer (Waters^®^, Milford, MA, USA) coupled to a Waters Acquity I-Class UPLC system (Waters^®^, Milford, MA, USA). HPLC-DAD-ELSD analysis was performed on a VWR Hitachi Chromaster system (VWR International, Allison Park, PA, USA) consisting of a 5430 diode array detector (VWR International, Allison Park, PA, USA), a 5310 column oven, and a 5110 pump combined in parallel with a VWR Evaporative Light Scattering Detector (ELSD 90, VWR International, Allison Park, PA, USA). Separations were performed on a semi-preparative C18 monolithic column (Onyx, 100 × 10 mm, Phenomenex) and an analytical Gemini^®^ 5µ C6-phenyl column (250 × 4.6 mm, 5 µ, Phenomenex). The enantiopurity of each purified compound was checked with a chiral cellulose-1 column (Lux 5µ, 250 × 4.6 mm, Phenomenex). For all HPLC analyses, two solvents, H_2_O + 0.1% FA (A) and acetonitrile + 0.1% FA (B), ULC/MS and HPLC grade, were used as the mobile phase. HR-ESIMS data were recorded in positive-ion mode on a micrOTOF II-high-performance TOF-MS system (Bruker^®^, Billerica, MA, USA) equipped with an electrospray ionization source. ^1^H and ^13^C NMR spectra (600 and 150 MHz, respectively) were recorded on a Bruker AV 600 spectrometer equipped with a triple resonance cryoprobe at 298 K (25 °C). The samples were dissolved in 300 μL of deuterated solvent using a 5.0 mm Shigemi tube. The residual solvent signals were used as internal references: *δ*_H_ 3.31/*δ*_C_ 49.0 ppm (MeOD) and tetramethylsilane (TMS) served as the internal standard. For compound **1**, the HSQC and HMBC spectra were run using non-uniform sampling and traditional planes (Supplementary Figures S6–S9). Optical rotations were measured in CHCl_3_ or in MeOH on a Jasco P-2000 polarimeter (Jasco, Pfungstadt, Germany) equipped with a sodium lamp (589 nm). The 3D structures of compounds **1**–**3** were obtained by using ChemBio3D Ultra 12.0 software (PerkinElmer, Waltham, MA, USA).

### 4.2. Sponge Material

The yellow sponge *C. celata* (Family Clionidae) was collected by scuba diving (−15 m) from the west side of Hormuz Island (Persian Gulf) in June 2016. The sample was immediately frozen at −20 °C and identified through a scanning optical microscope, skeletal slides, and dissociated spicule mounts [40]. A voucher specimen (De/Cl120) has been lodged with the Persian Gulf and Oman Sea Ecological Center.

### 4.3. Extraction and Isolation

The sponge *C. celata* (30 g dry wt.) was milled and desalted by extracting with Milli-Q Water (3 × 150 mL, 24 h) at room temperature. The sponge residue was successively extracted first with MeOH (3 *×* 150 mL, 48 h) and then with DCM (3 *×* 150 mL, 48 h) under the same conditions (under agitation at room temperature). The MeOH and DCM extracts were combined and evaporated to dryness on a rotary evaporator to yield a yellowish oily organic extract (150 mg). Desalting was done by SPE cartridge C18 (ChromaBond^®^ C18 PAH, 6 mL, 2000 mg, 30/ PAK) by washing with Milli-Q Water (3 × 15 mL) and then with MeOH (3 × 15 mL) and finally with DCM (3 *×* 15 mL) at room temperature. The dried combination of the two parts dissolved in MeOH and DCM was prepared and subjected to semi-preparative HPLC in a system equipped with an Onyx Monolithic Semi-PREP C18 column (Onyx, 100 × 10 mm Phenomenex) with a step gradient of MeOH in H_2_O (0% to 100% MeOH in 30 min) to afford nine fractions (F1–F9). The yellowish oily fraction F3 (22 mg) was further fractionated on the same HPLC system, eluting isocratically with a H_2_O + 0.1% FA (A):MeCN + 0.1% FA (B) (80:20) mixture (flow rate of 2.5 mL/min) to obtain four subfractions (F3a–F3d). Subfraction F3b (11 mg) was further purified by RP-HPLC (analytical Gemini^®^ 5µ C6-phenyl column, 250 × 4.6 mm, 5 μ, Phenomenex) employing an isocratic mixture of H_2_O + 0.1% FA (A):MeCN + 0.1% FA (B) (75:25) at a flow rate of 1.5 mL/min to yield **1** (3.0 mg, *t*_R_ 8.3 min) and **2** (3.0 mg, *t*_R_ 9.5 min). The elution of subfraction F4 (2.2 mg) in the same system by a mixture of 70% A and 30% B isocratically for 25 min afforded **3** (0.5 mg, *t*_R_ 7.2 min). The enantiopurity of each purified compound was checked by HPLC-DAD-ELSD on a chiral cellulose-1 analytical column (Lux 5 µ, 250 × 4.6 mm, Phenomenex) using a gradient of MeCN (1% to 100% for 15 min at a flow rate of 1.5 mL/min).

Compound **1**: yellowish oil. [α]^20^_D_ +30.3 (*c* 0.1, CHCl_3_), [α]^20^_D_ +23.0 (*c* 0.1, MeOH); ^1^H NMR (CD_3_OD, 600 MHz) and ^13^C NMR (CD_3_OD, 150 MHz), Table 1 and Table 2; HR-ESIMS *m*/*z* [M + H]^+^ 475.3285 (calculated for C_26_H_43_N_4_O_4_ 475.3284).

Compound **2**: yellowish oil. [α]^20^_D_ +21.3 (*c* 0.1, CHCl_3_); ^1^H NMR (CD_3_OD, 600 MHz) and ^13^C NMR (CD_3_OD, 150 MHz), Table 1 and Table 2; HR-ESIMS *m*/*z* [M + H]^+^ 489.3443 (calculated for C_27_H_45_N_4_O_4_ 489.3441).

Compound **3**: yellowish oil. [α]^20^_D_ +15.3 (*c* 0.1, CHCl_3_); ^1^H NMR (CD_3_OD, 600 MHz) and ^13^C NMR (CD_3_OD, 150 MHz), Table 1 and Table 2; HR-ESIMS *m*/*z* [M + H]^+^ 419.2552 (calculated for C_23_H_35_N_2_O_5_ 419.2546).

### 4.4. UPLC-QToF-MS/MS Analyses

The crude extract was analyzed in an ACQUITY UPLC I-Class System coupled to a Xevo G2-XS QToF Mass Spectrometer (Waters^®^, Milford, MA, USA), which was equipped with an electrospray ionization (ESI) source operating with a positive polarity at a mass range of *m*/*z* 50–1600 Da. The sample was prepared at a concentration of 0.1 mg/mL in methanol and filtered through a syringe filter (0.2 μm PTFE, Carl Roth, Karlsruhe, Germany), and then 2 µL of the sample was injected into the system. The compounds were separated using a binary LC solvent system controlled by MassLynx^®^ (version 4.1) to analyze MS and MS^2^ data. The separation was done by an Acquity UPLC HSS T3 column (high-strength silica C18, 1.8 µm, 100 × 2.1 mm I.D., Waters^®^) at 40 °C and eluted with H_2_O + 0.1% FA (A) and MeCN + 0.1% FA (B), ULC/MS grade, at a flow rate of 0.6 mL/min with the following gradient: initial, 1%–100% B, 0–12 min; 100% B, 12–13 min; and a column reconditioning phase until 15 min. ESI was set up as follows: source temperature 150 °C, capillary voltage 0.8 kV, sample cone voltage 40.0 V, desolvation temperature 550 °C, cone gas flow 50 L/h, and desolvation gas flow 1200 L/h. The MS^2^ settings were maintained at 30 eV of collision energy (CE).

### 4.5. Molecular Networking

The data of UPLC-HRMS/MS of the crude extract were used to create molecular networks. The output of UPLC-HRMS/MS was converted to mzXML format using MSConvert software. The converted data were processed by MZmine 2.53 [17] and then uploaded to the Global Natural Products Social molecular networking platform (http://gnps.ucsd.edu) using FileZilla (https://filezilla-project.org/, accessed on 3 September 2019) to create a network on the online workflow at the GNPS [18]. The MS^2^ spectra within ± 17 Da of the precursor *m*/*z* were removed to filter for the data and filtered by selecting only the top six peaks in the ± 50 Da window throughout the spectrum. The network of all data was achieved with MS-Network with an MS^2^ fragment ion tolerance of 0.02 Da and a parent mass tolerance of 0.02 Da to create consensus spectra, while those including less than two spectra of the consensus spectra were deleted. The network was created with a cosine score above 0.6 and more than three matched peaks. Moreover, edges between two nodes were held in the network if and only if each node arrived in the other’s top 10 most similar nodes. The database library of the GNPS was applied to filter the input data via library spectra due to similarities in the database. The input data must follow at least six match peaks with a score above 0.7 in the library spectra to be selected as a matched output spectrum. Cytoscape (ver. 3.7.1, provided by the U.S. National Institute of General Medical Sciences (NIGMS) under award number R01 GM070743) was used to visualize the output of molecular networking data [41].

### 4.6. Antimicrobial Activity

The activity of the crude extract and purified compounds was measured in 96-well plates with an effective concentration of 100 µg/mL. The tested samples were prepared as 20 mg/mL stock solutions in DMSO and then diluted with a medium and subjected to the test pathogens in 96-well plates. The bacteria were grown in TSB medium (0.5% NaCl, 1.2% tryptic soy broth), except for *E. faecium*, which was cultivated in M92 medium (3% trypticase soy broth, pH 7.0–7.2, 0.3% yeast extract). *C. neoformans* and *C. albicans* were cultivated in M186 (0.5% peptone from soymeal, 1% glucose, 0.3% malt extract, 0.3 yeast extract), and *C. albicans* was grown in M186/3 (0.1% malt extract, 0.3% glucose, 0.17% peptone from soymeal, 0.1% yeast extract). All microorganisms were purchased from Leibniz Institute DSMZ, Braunschweig, Germany, with strain numbers DSM 20477 (*E. faecium*), DSM 18827 (MRSA), DSM 1576 (*E. coli*), DSM 1386 (*C. albicans*), and DSM 6973 (*C. neoformans*). Overnight cultures of the test organisms were adjusted to an optical density of 600 nm and dilution of 0.01–0.03. Then, 200 µL of cell suspension cultures were added to each well. The microplates were incubated for 5 h at 37 °C and 200 rpm in all cases except for *C. neoformans*, which was incubated at 28 °C and 200 rpm for 7 h, and *E. faecium*, which was incubated for 5 h at 37 °C without shaking. The inhibitory effects were detected by adding 10 µL of a resazurin solution (0.3 mg/mL phosphate-buffered saline) to each well and then incubating again for 5–30 min. The fluorescence signal (560 nm/590 nm) was measured by the microfluidic instrument (Tecan Infinite M200), while for *E. faecium*, the pH indicator bromocresol purple was used to determine the acidification caused by growing, and for *R. solanacearum* and *C. neoformans*, the optical density at 600 nm after the incubation time was recorded using the microplate reader. The IC_50_ values were calculated by Excel to determine the concentration that shows 50% inhibition of viability.

### 4.7. Cytotoxic Activity

The cytotoxic activity against cancer cell lines was assessed by monitoring the metabolic activity at 37 °C under a humidified atmosphere and 5% CO_2_ in the CellTiterBlue Cell Viability Assay (Promega, Mannheim, Germany). The cultivation of HaCaT (CLS Cell Lines Service, Eppelheim, Germany), HT29, and Hep G2 (Leibniz Institute DSMZ, Braunschweig, Germany) cells was carried out in RPMI 1640 medium (Life Technologies, Darmstadt, Germany) supplemented with 10% fetal bovine serum, 100 U/mL of penicillin, and 100 mg/mL of streptomycin at 37 °C and 5% CO_2_. The cultivation of A549 cells (CLS Cell Lines Service, Eppelheim, Germany) was carried out in DMEM:Ham’s F12 medium (1:1) supplemented with 15 mM HEPES, and that of A375 and HCT116 cells (CLS Cell Lines Service, Eppelheim, Germany) was carried out in DMEM supplemented with 4.5 g/L of D-glucose and 110 mg/L of sodium pyruvate. For bioactivity tests, the seeding of cells was performed in 96-well plates at a concentration of 10,000 cells per well. For the experimental procedure, the tested samples were prepared as a 20 mg/mL stock solution in DMSO. After 24 h of cultivation, 100 µL of fresh medium containing the tested samples was replaced with the medium in the cells and again cultivated for 24 h at 37 °C. The anticancer drug tamoxifen was used as the positive control, while the growth media and 0.5% DMSO were used as negative controls. Further, the assay was performed according the CellTiterBlue Cell Viability Assay protocol described by the manufacturer (Promega, Mannheim, Germany). The inhibition rates were computed from fluorescence measurements taken with the Tecan Infinite M200 microplate reader (Tecan, Crailshaim, Germany) at an excitation wavelength of 560 nm and an emission wavelength of 590 nm. Calculation of IC_50_ values was done by Excel to determine the concentration that shows 50% inhibition of viability.

## Figures and Tables

**Figure 6:**
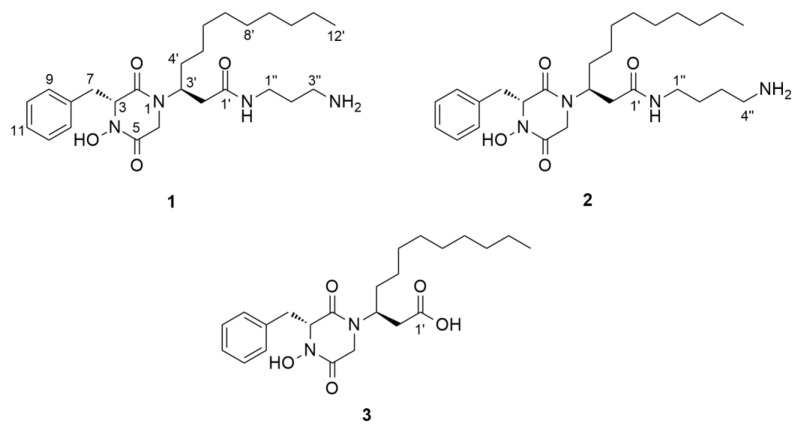
Chemical structure of compounds **1**–**3**.

**Table 1:** ^1^H NMR (600 MHz) data of compounds **1**–**3** in CD_3_OD.

Position	*δ*_H_, Mult. (*J* in Hz)		
	1	2	3
1 (N)	-	-	-
2	-	-	-
3	4.48 (m)	4.42 (m)	4.50 (m)
4 (N)	-	-	-
5	-	-	-
6	3.47 (d 16.8) 2.51 (d 16.8)	3.47 (d 16.6) 2.60 (d 16.6)	3.52 (d 17.3) 2.41 (d 17.3)
7	3.18 (m) 3.45 (m)	3.15 (dd 13.8, 2.7) 3.47 (m)	3.18 (dd 13.8, 2.7) 3.43 (m)
8	-	-	-
9	7.20 (d 7.1)	7.20 (d 7.1)	7.19 (m)
10	7.30 (m)	7.28 (m)	7.30 (m)
11	7.30 (m)	7.28 (m)	7.31 (m)
12	7.30 (m)	7.28 (m)	7.30 (m)
13	7.20 (m)	7.20 (m)	7.19 (m)
1′	-	-	-
2′	2.16 (dd 14.2, 7.7) 2.41 (m)	2.16 (dd 14.2, 6.6) 2.60 (m)	2.15 (dd 15.16, 7.23) 2.27 (m)
3′	4.24 (br)	3.93 (br)	4.57 (m)
4′	1.51 (m) 1.57 (m)	1.53 (m) 1.63 (m)	1.48 (m) 1.57 (m)
5′	1.08 (m)	1.07 (m)	1.10 (m)
6–′9′	~1.27 (m)	~1.29 (m)	~1.27 (m)
10′	1.28 (m)	1.29 (m)	1.28 (m)
11′	1.32 (m)	1.32 (m)	1.32 (m)
12′	0.91 (t 7.1)	0.91 (t 7.1)	0.90 (t 7.1)
1″	3.17 (m) 3.32 (m)	3.02 (m) 3.37 (m)	-
2″	1.77 (p 7.2)	1.54 (m)	-
3″	2.85 (t 7.2)	1.55 (m)	-
4″	-	2.87 (m)	-

**Table 2:** ^13^C NMR (150 MHz) data of compounds **1**–**3** in CD_3_OD.

Position	*δ*_C_, Type		
	1	2	3
1 (N)	-	-	-
2	167.3 (C)	167.3 (C)	166.9 (C)
3	66.7 (CH)	67.0 (CH)	66.3 (CH)
4 (N)	-	-	-
5	162.2 (C)	161.3 (C)	163.1 (C)
6	47.4 (CH_2_)	n.o. ^1^	45.7 (CH_2_)
7	36.4 (CH_2_)	36.4 (CH_2_)	36.4 (CH_2_)
8	136.1 (C)	136.3 (C)	135.8 (C)
9	131.5 (CH)	131.5 (CH)	131.4 (CH)
10	129.7 (CH)	129.7 (CH)	129.8 (CH)
11	128.7 (CH)	128.7 (CH)	128.8 (CH)
12	129.7 (CH)	129.7 (CH)	129.8 (CH)
13	131.5 (CH)	131.5 (CH)	131.4 (CH)
1′	173.2 (C)	172.9 (C)	175.8 (C)
2′	39.1 (CH_2_)	39.2 (CH_2_)	38.9 (CH_2_)
3′	58.1 (CH)	n.o. ^1^	n.o. ^1^
4′	31.4 (CH_2_)	31.6 (CH_2_)	31.4 (CH_2_)
5′	27.2 (CH_2_)	27.4 (CH_2_)	27.1 (CH_2_)
6′–9′	30.2-30.06 (CH_2_)	30.2-30.06 (CH_2_)	30.2-30.06 (CH_2_)
10′	33.0 (CH_2_)	33.0 (CH_2_)	33.0 (CH_2_)
11′	23.7 (CH_2_)	23.7 (CH_2_)	23.7 (CH_2_)
12′	14.4 (CH_3_)	14.4 (CH_3_)	14.4 (CH_3_)
1″	37.1 (CH_2_)	39.7 (CH_2_)	-
2″	29.8 (CH_2_)	27.7 (CH_2_)	-
3″	38.6 (CH_2_)	26.5 (CH_2_)	-
4″	-	40.4 (CH_2_)	-

^1^ n.o.: not observed.

**Table 3:** Bioactivity of compounds **1** and **2**.

Sample	IC_50_ Values (in µM)							
	*E*. *faecium*	MRSA	*E*. *coli*	*C. albicans*	*C. neoformans*	A375	HCT116	HaCaT
Compound **1**	19.6	46.2	159.1	116.0	22.6	43.2	59.9	50.0
Compound **2**	36.3	44.7	152.9	125.0	28.1	37.9	39.5	37.5
Positive control	0.57	4.6	4.7	2.3	0.2	0.2	0.4	42.1

Positive controls: MRSA and *E*. *coli*: chloramphenicol; *E*. *faecium*: ampicillin; *C. albicans*: nystatin; *C. neoformans*: amphotericin B; A375, HCT116, and HaCaT: doxorubicin.

## Data Availability

Not applicable.

## Supplementary Materials

The following are available online at https://www.mdpi.com/article/10.3390/md19080439/s1: a table showing the putative identification of compounds in the global molecular network of the crude sponge extract, a global molecular network of the crude extract of *C. celata*, UPLC-HRMS chromatogram of the crude extract, and 1D and 2D NMR and HR-ESIMS spectra of compounds **1**–**3**.
