# Supplementary material for: Molecular Networking-Guided Isolation of New Etzionin-Type Diketopiperazine Hydroxamates from the Persian Gulf Sponge Cliona celata
Source: Mar Drugs. 2021 Jul 31;19(8):439. doi: 10.3390/md19080439 (PMC8402168; doi:10.3390/md19080439)

Supporting Information

Molecular Networking-Guided Isolation of New Etzionin-Type Diketopiperazine Hydroxamates from the Persian Gulf Sponge Cliona *celata*

Reza Mohsenian Kouchaksaraee[1], Fengjie Li[1], Melika Nazemi,[2] Mahdi Moridi Farimani [3] and Deniz Tasdemir [1,4,*]

[1] GEOMAR Centre for Marine Biotechnology (GEOMAR-Biotech), Research Unit Marine Natural Products Chemistry, GEOMAR Helmholtz Centre for Ocean Research Kiel, Am Kiel-Kanal 44, 24106 Kiel, Germany; rmohsenian@geomar.de; fli@geomar.de

[2] Persian Gulf and Oman Sea Ecological Center, Iranian Fisheries Science Research Institute, Agricultural Research, Education and Extension Organization (AREEO), 7916793165 Bandar Abbas, Iran; melikanazemi@pgoseri.ac.ir

[3] Department of Phytochemistry, Medicinal Plants and Drugs Research Institute, Shahid Beheshti University, G. C., Evin, 1983969411 Tehran, Iran; m_moridi@sbu.ac.ir

[4] Faculty of Mathematics and Natural Sciences, Kiel University, Christian-Albrechts-Platz 4, 24118 Kiel, Germany

* Correspondence: dtasdemir@geomar.de; Tel.: +49-431-6004430

Content **Page No.**

Table S1. Putative identification of the compounds (clusters A and B) in the global molecular network of the crude extract of *Cliona celata* 3

Figure S1. Global molecular network of the crude extract of *Cliona celata* 4

Figure S2. UPLC-HRMS chromatogram of the crude extract from *Cliona celata* 5

Figure S3. ^{1}H NMR spectrum of the fraction F3 from *Cliona celata* (600 MHz, CD_3OD) 5

Figure S4. ^{1}H NMR spectrum of compound **1** (TFA salt, 600 MHz, CD_3OD) 6

Figure S5. ^{13}C NMR spectrum of compound **1** (TFA salt, 150 MHz, CD_3OD) 6

Figure S6. HSQC spectrum of compound **1** (TFA salt, 600 MHz, CD_3OD, non-uniform sampling) 7

Figure S7. HSQC spectrum of compound **1** (TFA salt, 600 MHz, CD_3OD, traditional planes) 7

Figure S8. HMBC spectrum of compound **1** (TFA salt, 600 MHz, CD_3OD, non-uniform sampling) 8

Figure S9. HMBC spectrum of compound **1** (TFA salt, 600 MHz, CD_3OD, traditional planes) 8

Figure S10. COSY spectrum of compound **1** (TFA salt, 600 MHz, CD_3OD) 9

Figure S11. NOESY spectrum of compound **1** (TFA salt, 600 MHz, CD_3OD) 9

Figure S12. HR-ESIMS spectrum of compound **1** 10

Figure S13. ^{1}H NMR spectrum of compound **2** (TFA salt, 600 MHz, CD_3OD) 11

Figure S14. ^{13}C NMR spectrum of compound **2** (TFA salt, 150 MHz, CD_3OD) 11

Figure S15. HSQC spectrum of compound **2** (TFA salt, 600 MHz, CD_3OD) 12

Figure S16. HMBC spectrum of compound **2** (TFA salt, 600 MHz, CD_3OD) 12

Figure S17. COSY spectrum of compound **2** (TFA salt, 600 MHz, CD_3OD) 13

Figure S18. NOESY spectrum of compound **2** (TFA salt, 600 MHz, CD_3OD) 13

Figure S19. HR-ESIMS spectrum of compound **2** 14

Figure S20. ^{1}H NMR spectrum of compound **3** (TFA salt, 600 MHz, CD_3OD) 15

Figure S21. ^{13}C NMR spectrum of compound **3** (TFA salt, 150 MHz, CD_3OD) 15

Figure S22. HSQC spectrum of compound **3** (TFA salt, 600 MHz, CD_3OD) 16

Figure S23. HMBC spectrum of compound **3** (TFA salt, 600 MHz, CD_3OD) 16

Figure S24. COSY spectrum of compound **3** (TFA salt, 600 MHz, CD_3OD) 17

Figure S25. NOESY spectrum of compound **3** (TFA salt, 600 MHz, CD_3OD) 17

Figure S26. HR-ESIMS spectrum of compound **3** 18

Table S1. Putative identification of the compounds (cluster A and B) in the global molecular network of the crude extract of *Cliona celata.*

No.	t_R (min)	Parent mass *m/z*	Cluster	Molecular formula of the *m/z* [M + H]$^+$	Δ ppm	Key MS^2 fragments	LoA*
1	5.43	459.332	A	$C_{26}H_{43}N_4O_3$	0.0	205.097; 238.217; 343.238; 385.249; 441.297	3
2	4.97	473.293	A	$C_{26}H_{41}N_4O_4$	0.4	221.093; 236.202; 357.213; 399.229; 456.887	3
3	5.42	475.315	A	$C_{26}H_{43}N_4O_4$	0.1	221.093; 252.233; 359.233; 401.244; 458.302	5
4	5.44	489.332	A	$C_{27}H_{45}N_4O_4$	0.2	221.093; 259.233; 359.233; 401.224; 471.333	5
5	6.03	491.305	A	$C_{26}H_{43}N_4O_5$	0.4	214.181; 238.218; 375.228; 417.240; 474.229	3
6	5.54	503.359	A	$C_{28}H_{47}N_4O_4$	0.4	221.093; 266.248; 359.233; 401.243; 485.347	3
7	5.86	503.359	A	$C_{28}H_{47}N_4O_4$	1.2	221.093; 266.249; 373.249; 415.259; 485.347	3
8	5.35	505.338	A	$C_{27}H_{45}N_4O_5$	-0.1	221.090; 238.216; 359.233; 401.240; 487.332	3
9	6.04	505.339	A	$C_{27}H_{45}N_4O_5$	0.6	214.180; 252.232; 375.229; 417.246; 487.237	3
10	5.28	637.373	A	$C_{32}H_{53}N_4O_9$	0.8	221.093; 238.277; 359.232; 401.245; 553.339	3
11	7.04	419.231	B	$C_{23}H_{35}N_2O_5$	0.6	181.159; 196.170; 221.093; 359.233; 401.244	5
12	7.15	403.235	B	$C_{23}H_{33}N_2O_4$	0.6	181.159; 205.096; 315.242; 343.237; 385.247	3

*: LoA (Level of Assignment); 1: Accurate mass matched to database-Tentative assignment, 2: Accurate mass matched to database and tandem MS spectrum matched to *in silico* fragmentation pattern, 3: Tandem MS spectrum matched to database or literature, 4: RT matched to standard compound, 5: MS/MS spectrum matched to standard compound.
Manual dereplication was performed considering the parent mass, biological source, retention time, elemental composition analysis, and predicated fragmentation patterns.
The MS^2 fragmentation pattern of a molecule was predicated on the Competitive Fragmentation Modeling for Metabolite Identification (CFM-ID) platform (http://cfmid.wishartlab.com) and compared with our experimental data.

Figure S1. Global molecular network of the crude extract of *Cliona celata*.

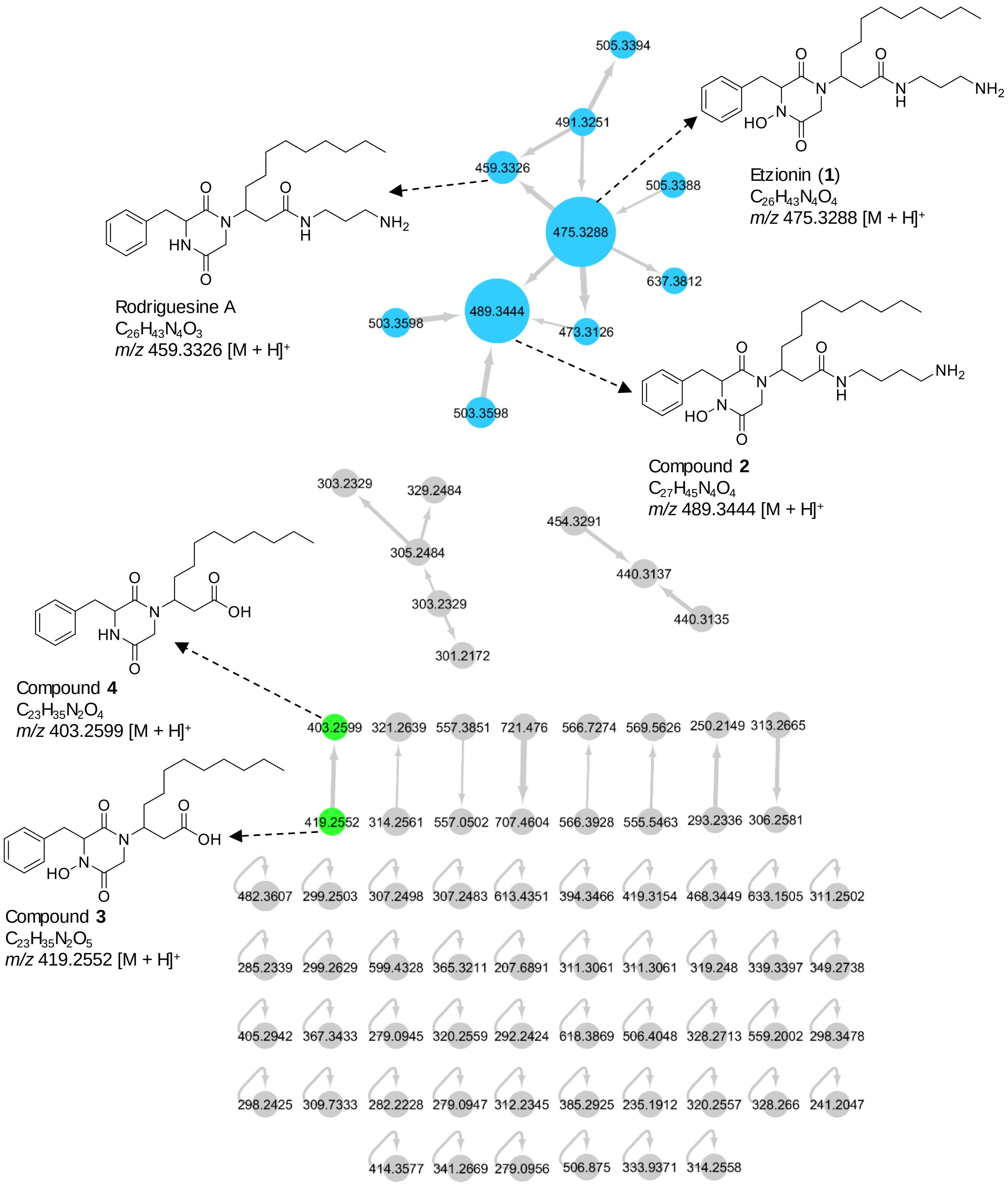

Figure S2. UPLC-HRMS chromatogram of the crude extract from *Cliona celata*.

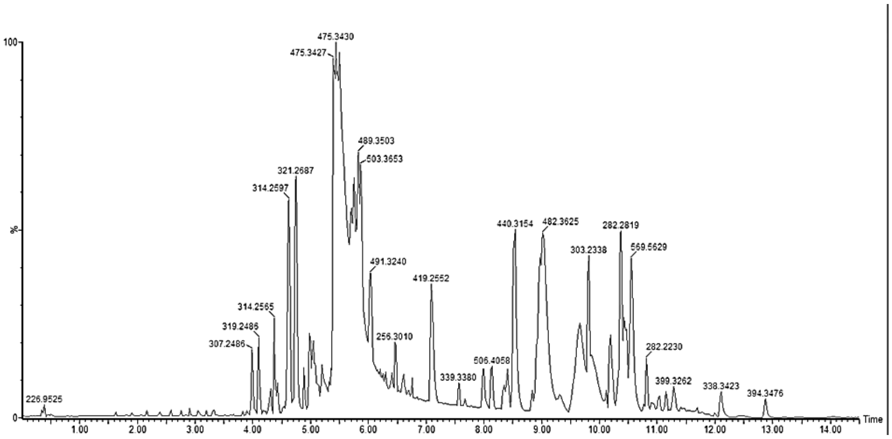

Figure S3. ^{1}H NMR spectrum of the fraction F3 from *Cliona celata* (600 MHz, CD_3OD)

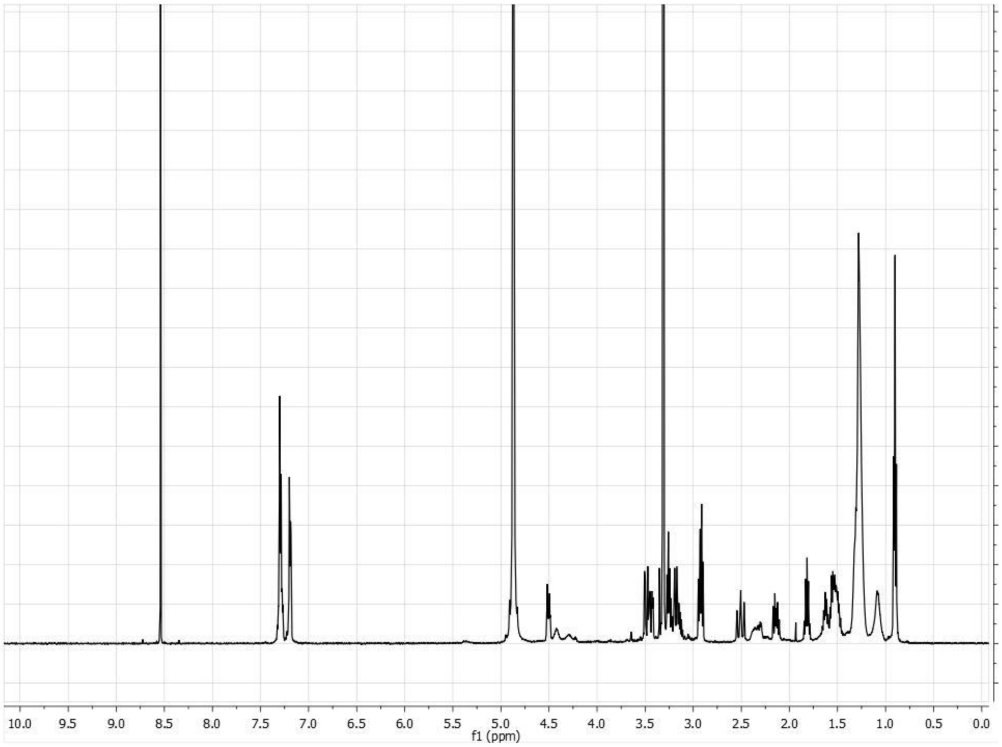

Figure S4. ^{1}H NMR spectrum of compound **1** (TFA salt, 600 MHz, CD_3OD).

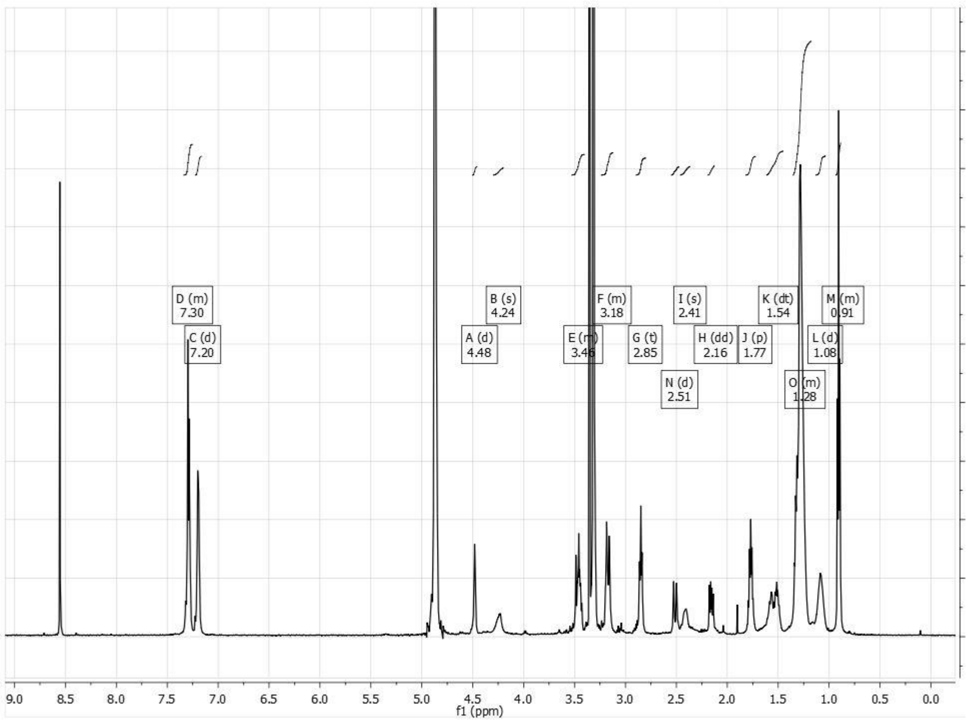

Figure S5. ^{13}C NMR spectrum of compound **1** (TFA salt, 150 MHz, CD_3OD)

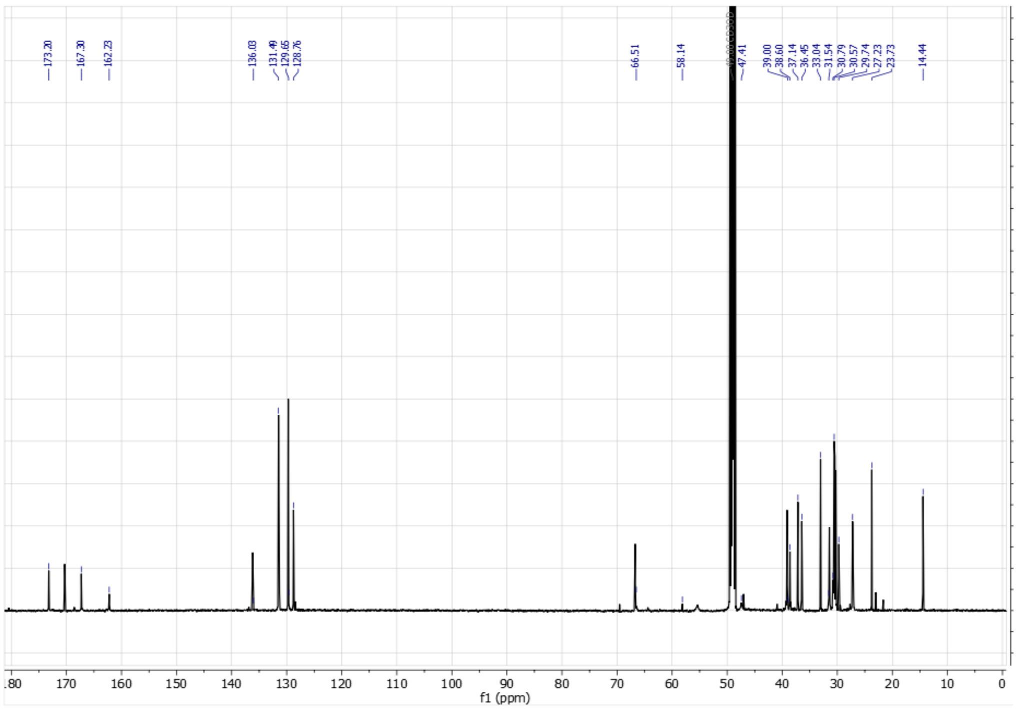

Figure S6. HSQC spectrum of compound **1** (TFA salt, 600 MHz, CD_3OD, non-uniform sampling).

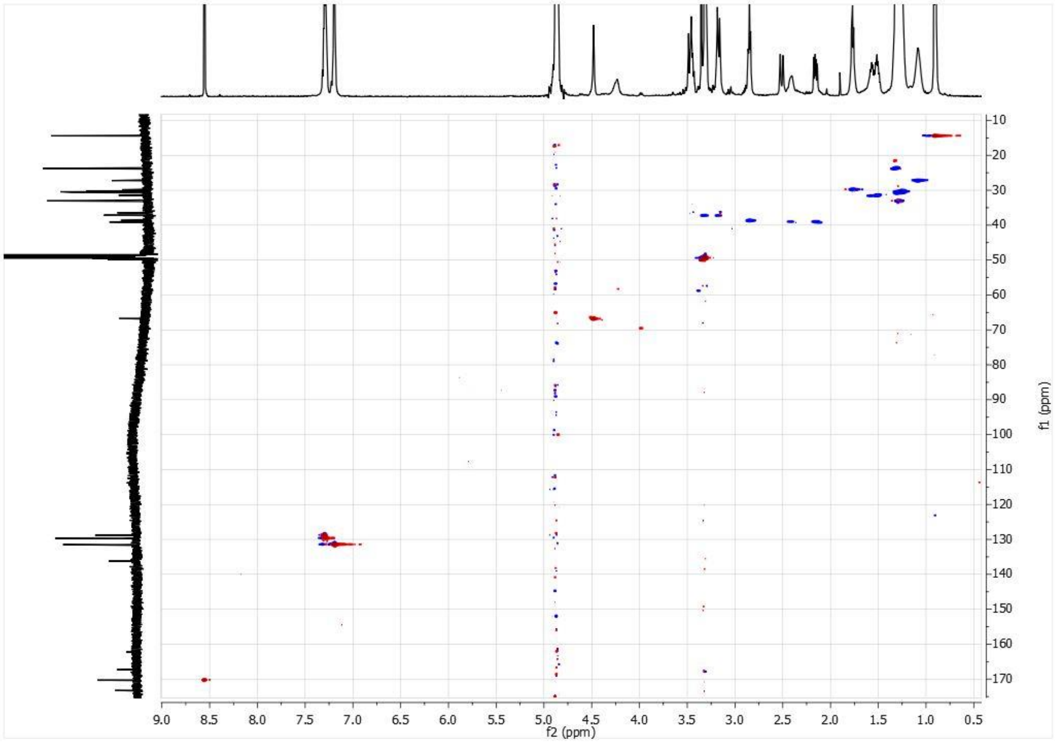

Figure S7. HSQC spectrum of compound **1** (TFA salt, 600 MHz, CD_3OD, traditional planes).

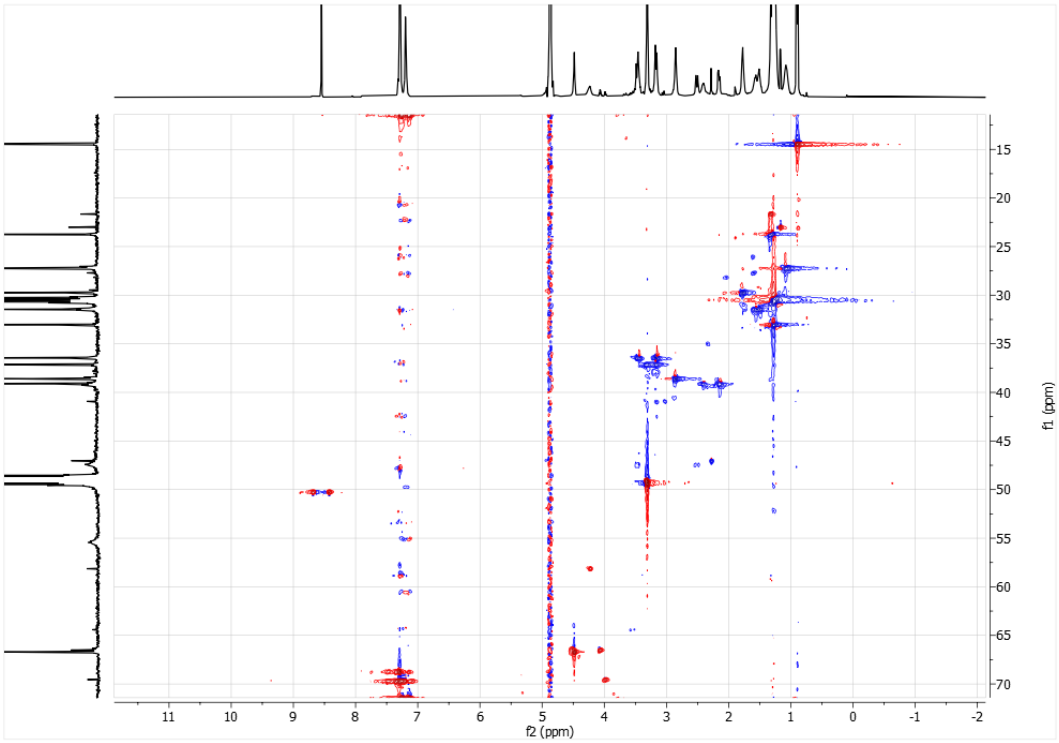

Figure S8. HMBC spectrum of compound **1** (TFA salt, 600 MHz, CD_3OD, non-uniform sampling).

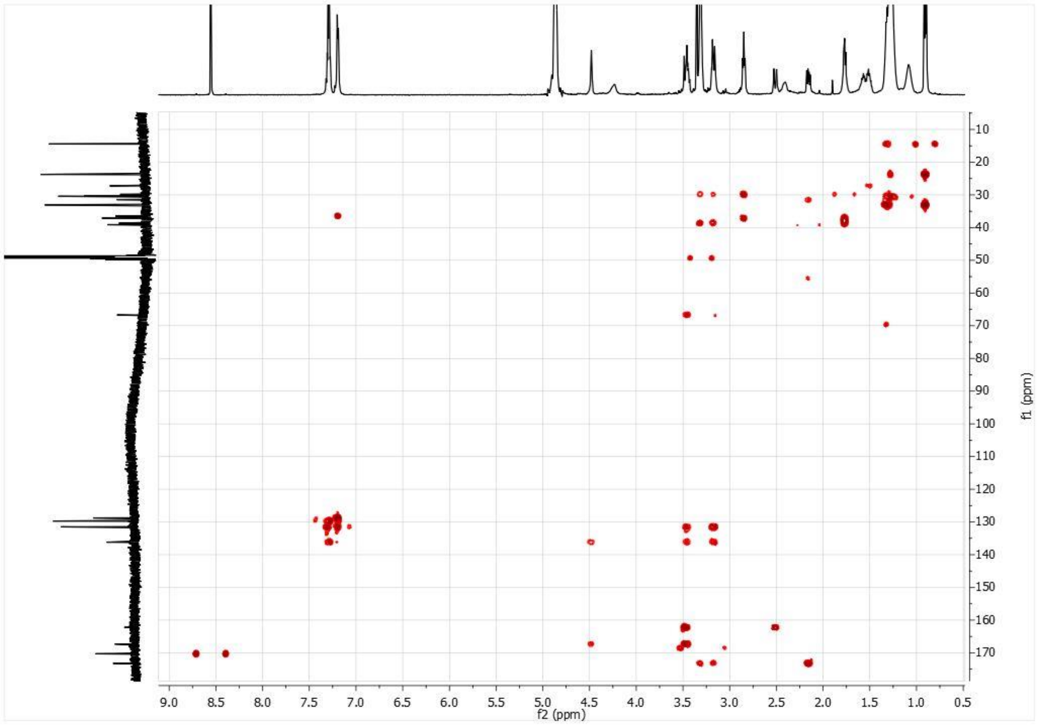

Figure S9. HMBC spectrum of compound **1** (TFA salt, 600 MHz, CD_3OD, traditional planes).

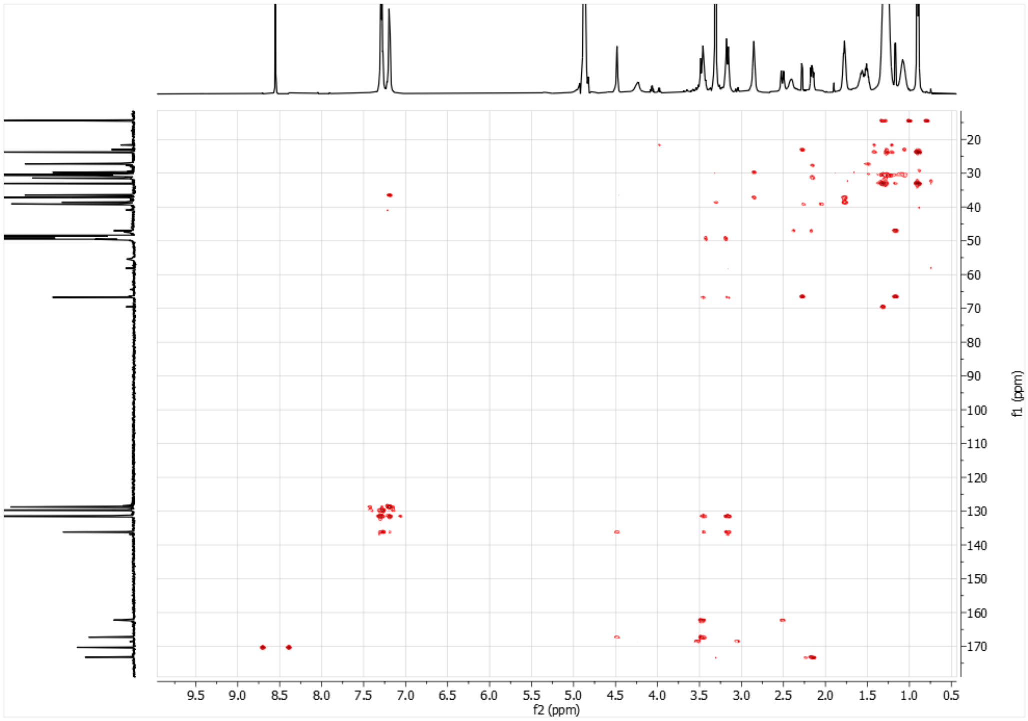

Figure S10. COSY spectrum of compound **1** (TFA salt, 600 MHz, CD_3OD).

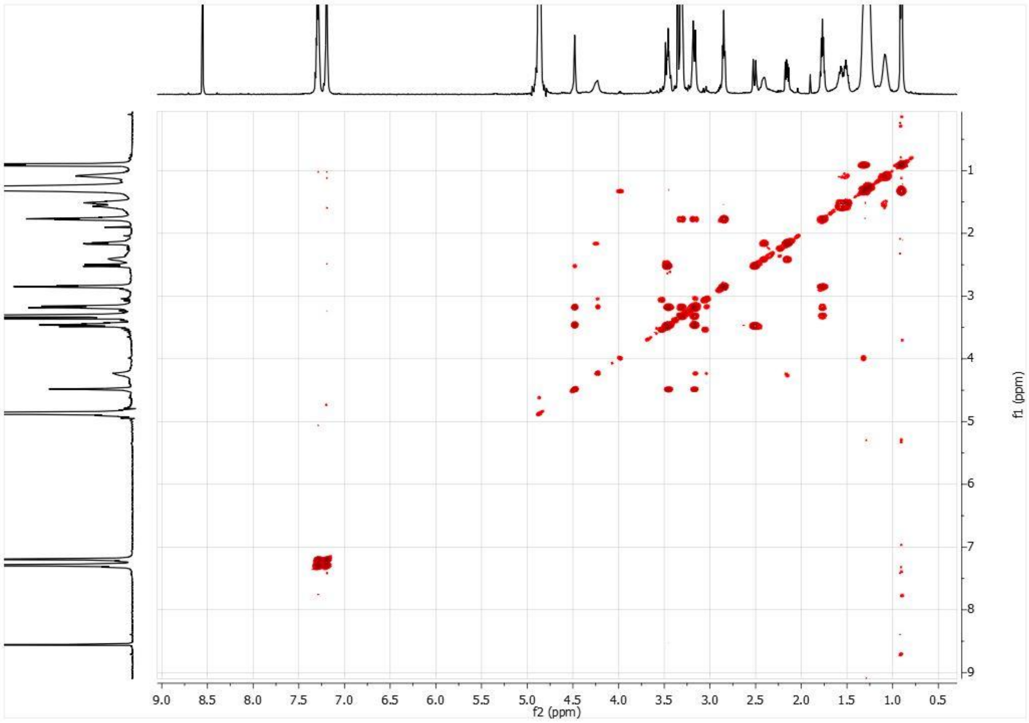

Figure S11. NOESY spectrum of compound **1** (TFA salt, 600 MHz, CD_3OD).

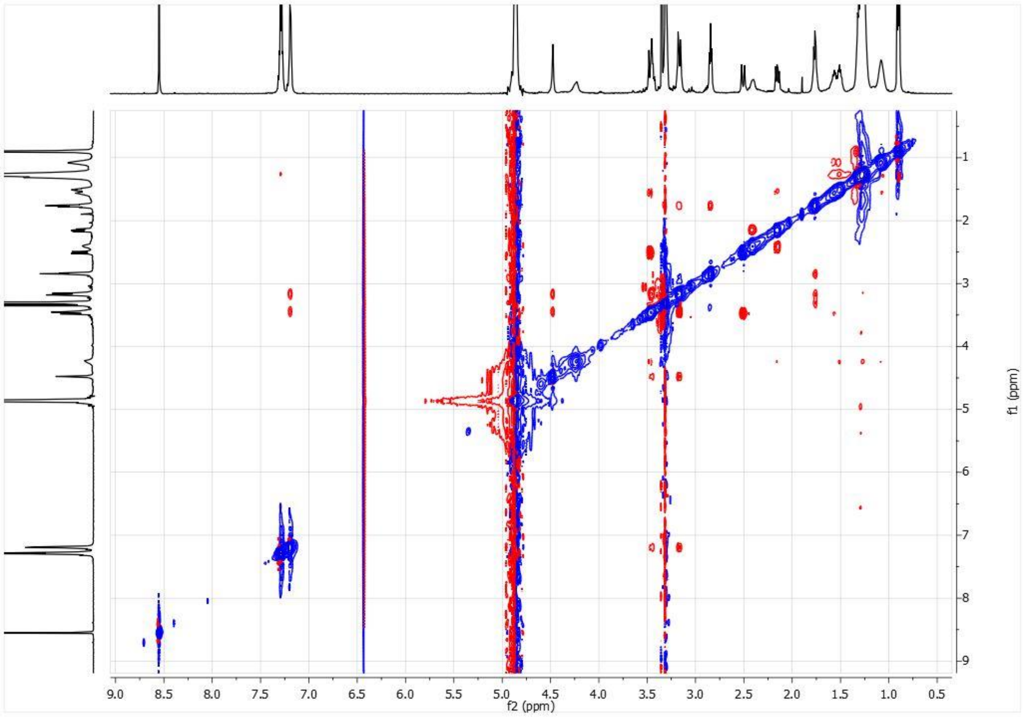

Figure S12. HR-ESIMS spectrum of compound **1**.

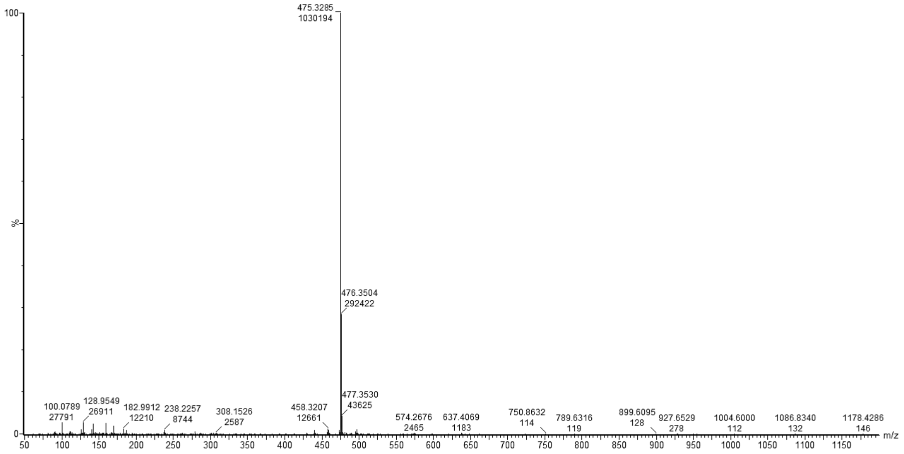

Figure S13. ^{1}H NMR spectrum of compound **2** (TFA salt, 600 MHz, CD_3OD).

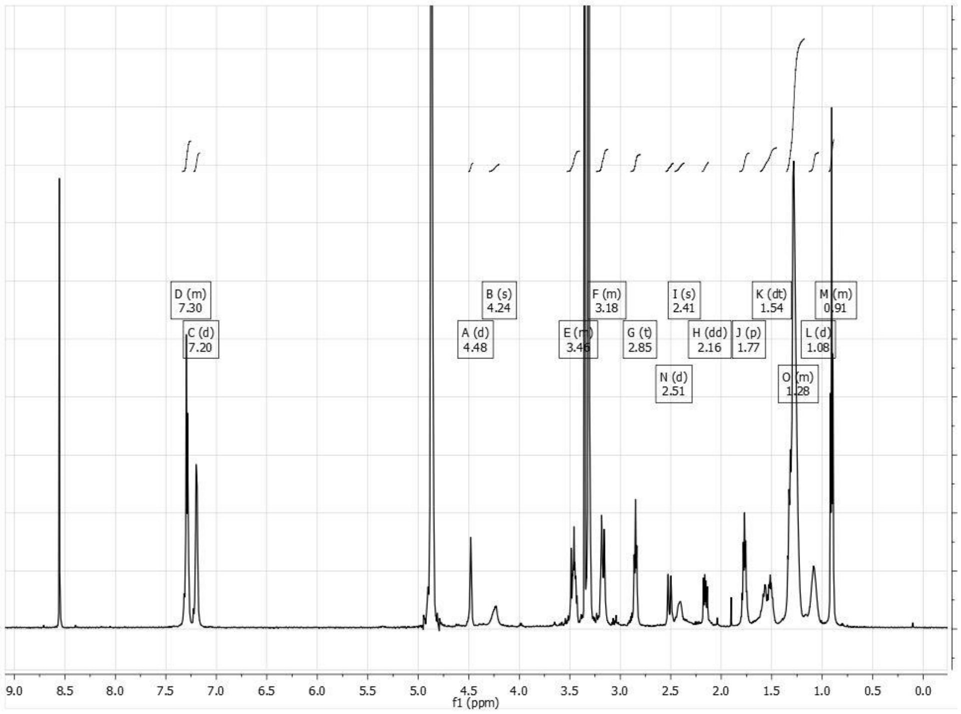

Figure S14. ^{13}C NMR spectrum of compound **2** (TFA salt, 150 MHz, CD_3OD).

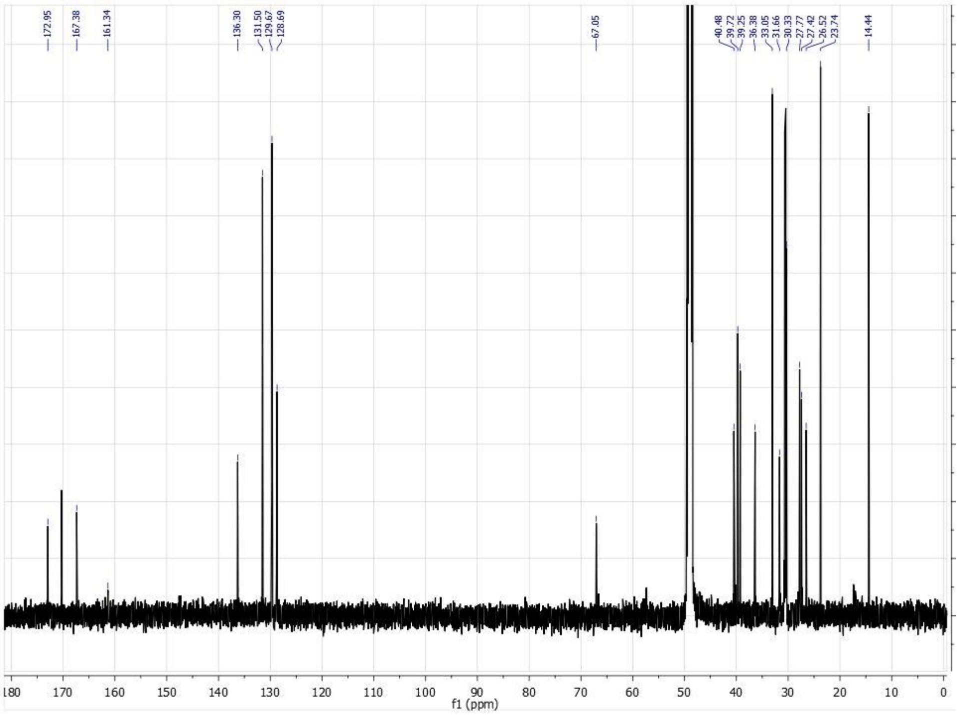

Figure S15. HSQC spectrum of compound **2** (TFA salt, 600 MHz, CD_3OD).

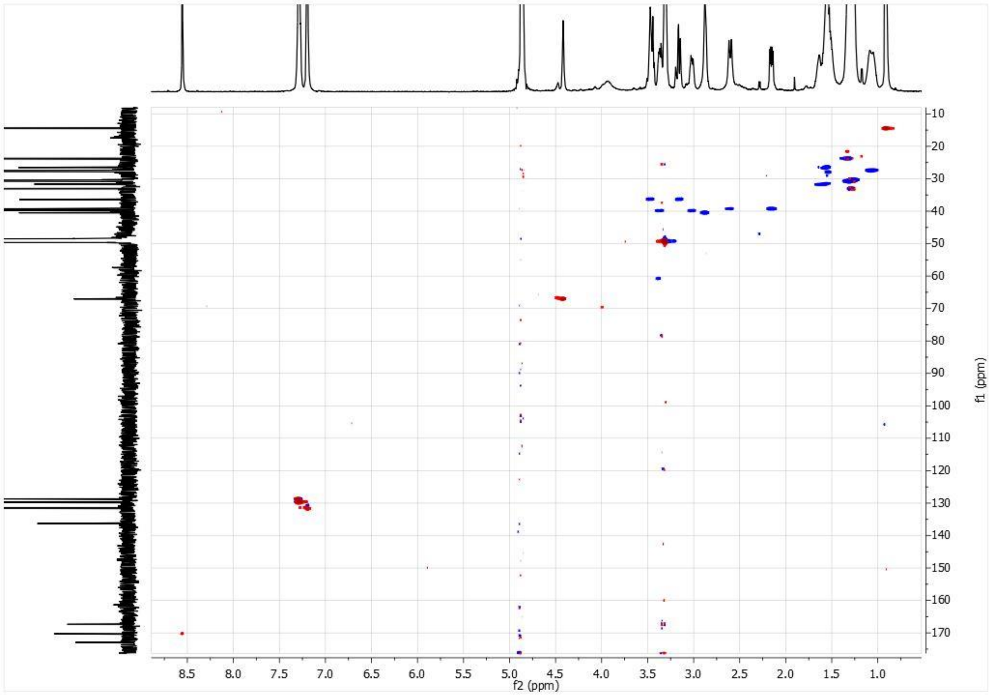

Figure S16. HMBC spectrum of compound **2** (TFA salt, 600 MHz, CD_3OD).

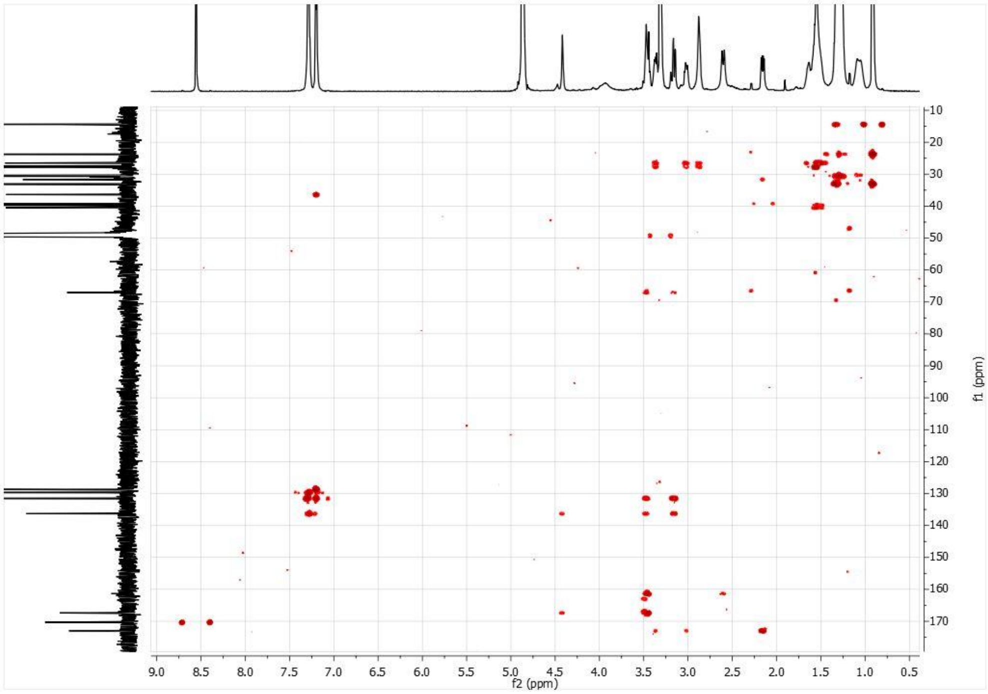

Figure S17. COSY spectrum of compound **2** (TFA salt, 600 MHz, CD_3OD).

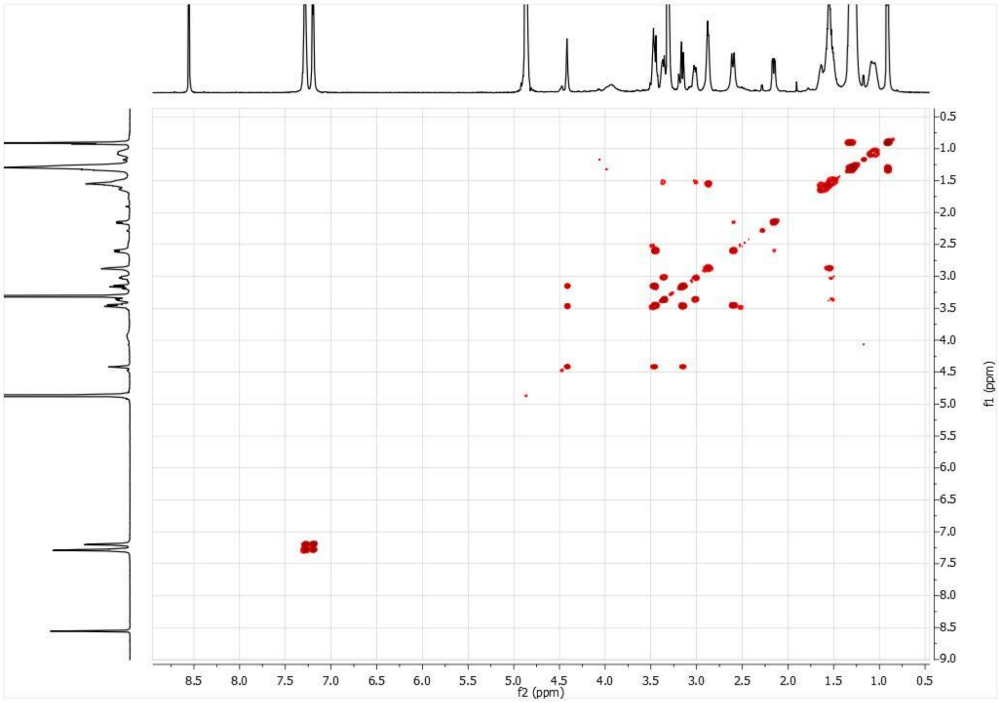

Figure S18. NOESY spectrum of compound **2** (TFA salt, 600 MHz, CD_3OD).

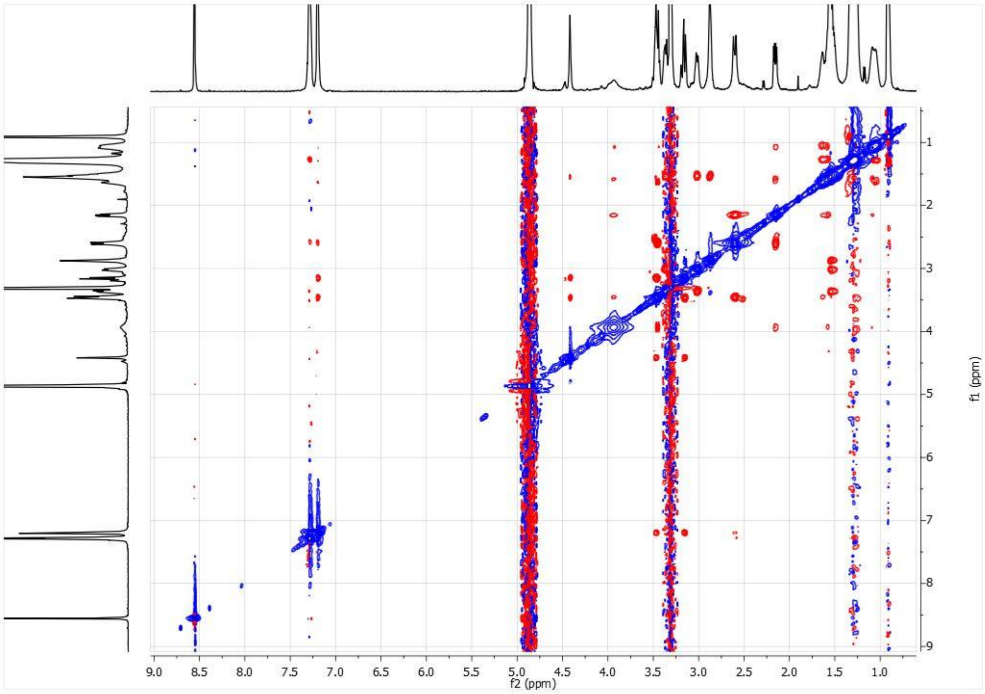

Figure S19. HR-ESIMS spectrum of compound **2**.

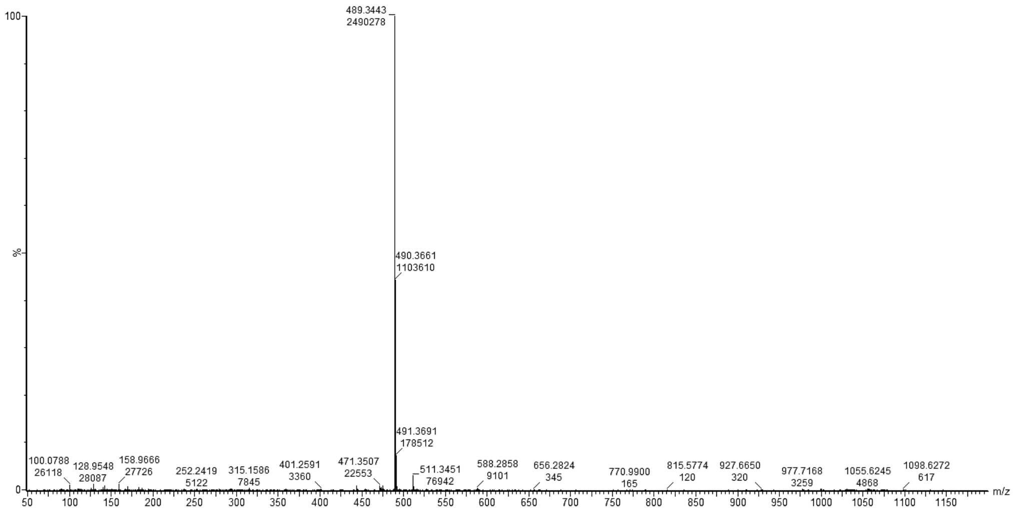

Figure S20. ^{1}H NMR spectrum of compound **3** (TFA salt, 600 MHz, CD_3OD).

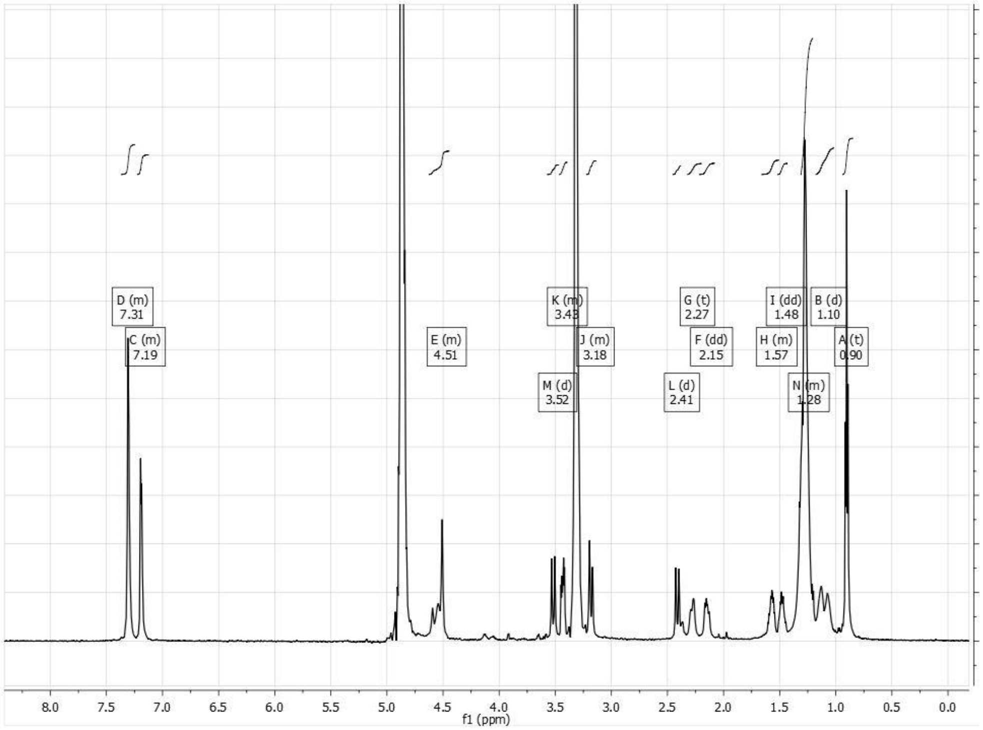

Figure S21. ^{13}C NMR spectrum of compound **3** (TFA salt, 150 MHz, CD_3OD).

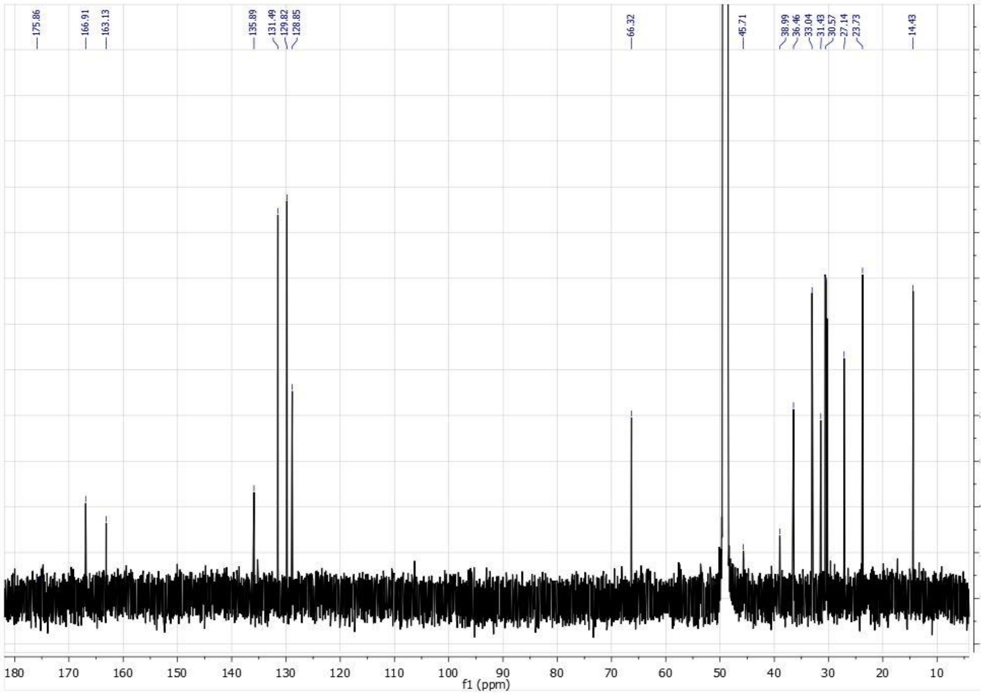

Figure S22. HSQC spectrum of compound **3** (TFA salt, 600 MHz, CD_3OD).

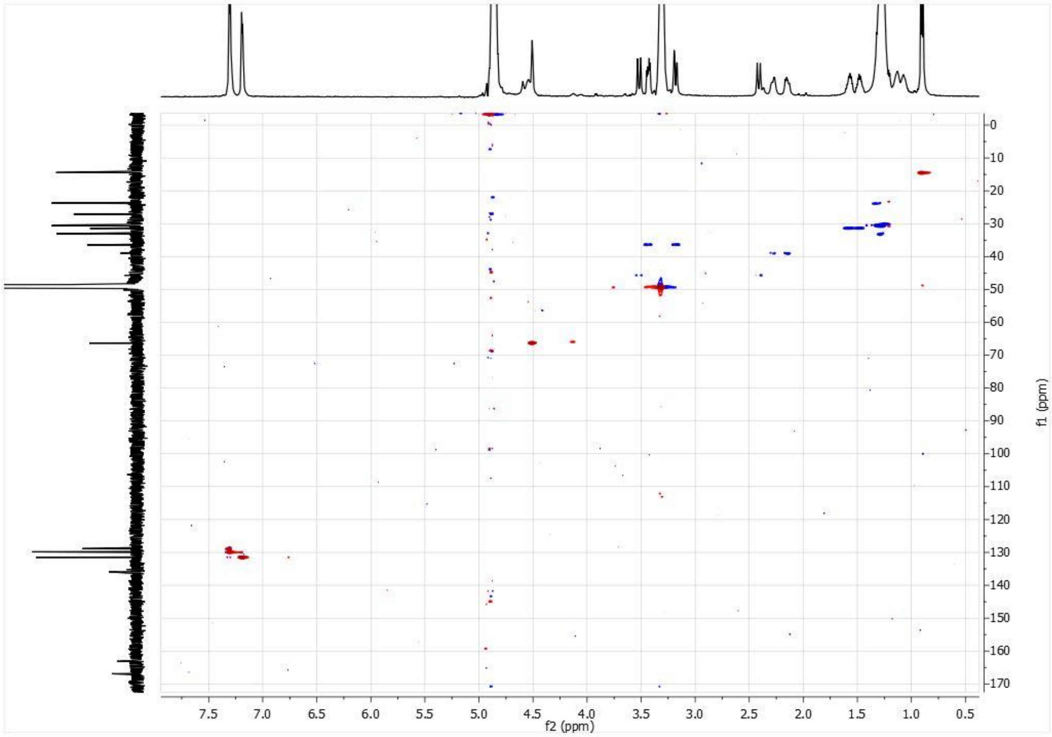

Figure S23. HMBC spectrum of compound **3** (TFA salt, 600 MHz, CD_3OD).

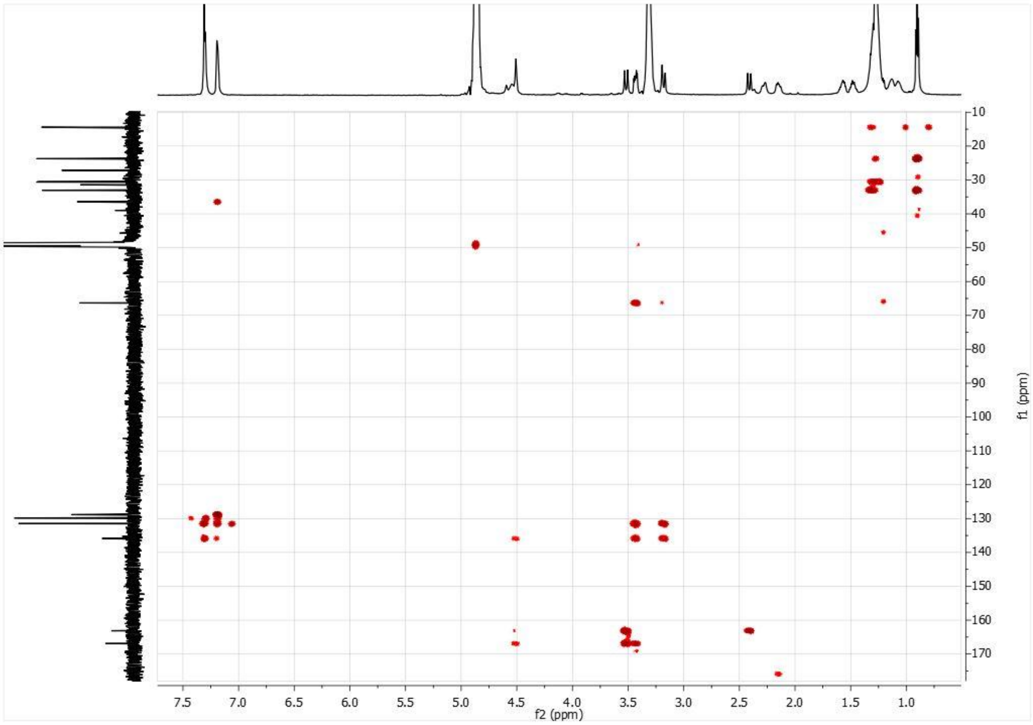

Figure S24. COSY spectrum of compound **3** (TFA salt, 600 MHz, CD_3OD).

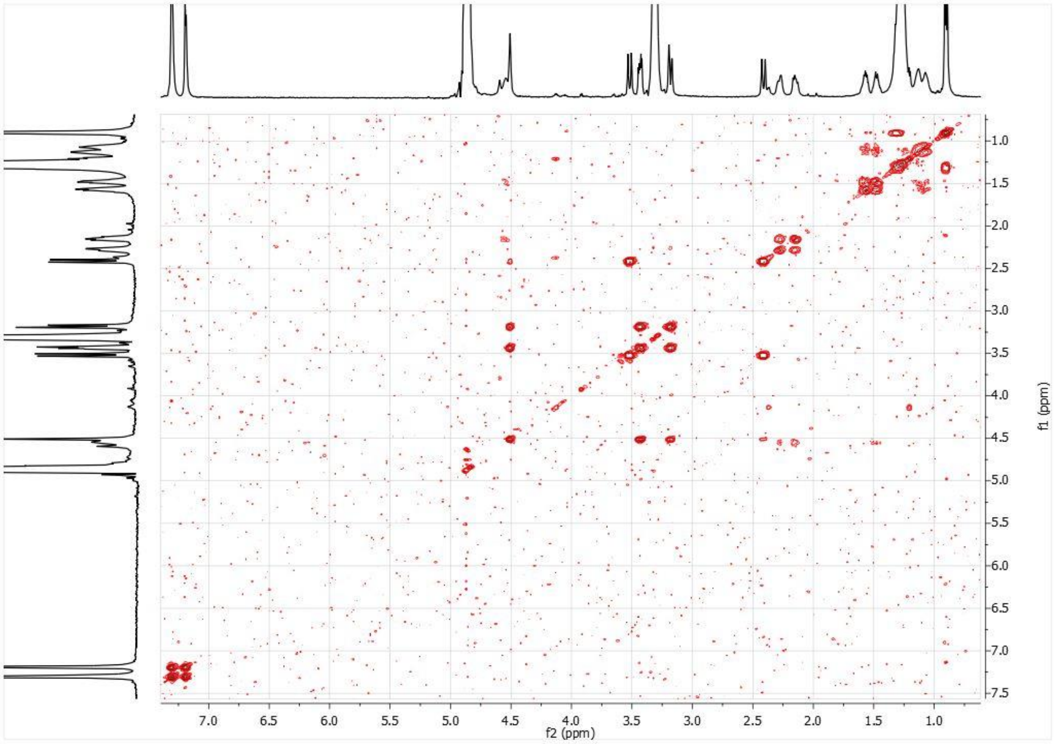

Figure S25. NOESY spectrum of compound **3** (TFA salt, 600 MHz, CD_3OD).

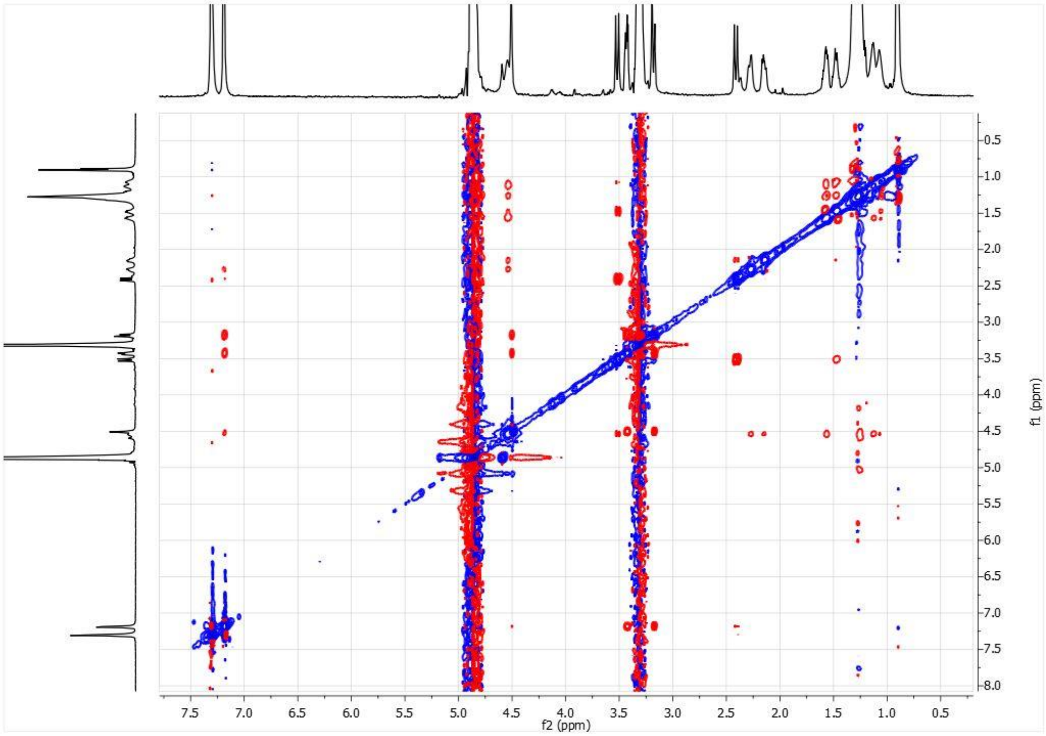

Figure S26. HR-ESIMS spectrum of compound **3**.

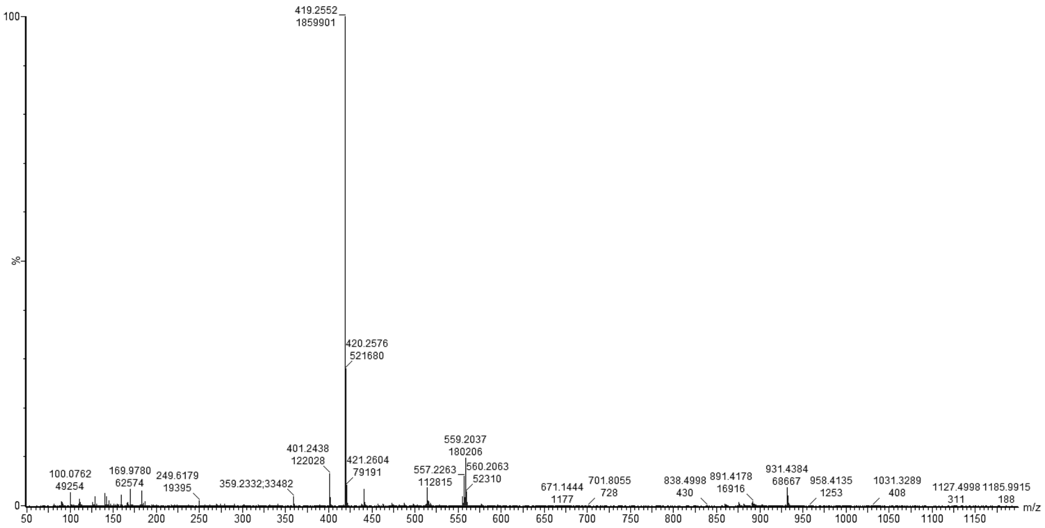

Supplement: Supplementary file 1 [file marinedrugs-19-00439-s001.zip › marinedrugs-1285160-supplementary.pdf]
